# Hierarchical dynamic coding coordinates speech comprehension in the human brain

**DOI:** 10.1073/pnas.2422097122

**Published:** 2025-10-17

**Authors:** Laura Gwilliams, Alec Marantz, David Poeppel, Jean-Rémi King

**Affiliations:** ^a^Department of Psychology, Stanford University, Stanford, CA 94305; ^b^Wu Tsai Neurosciences Institute, Stanford University, Stanford, CA 94305; ^c^Stanford Data Science, Stanford University, Stanford, CA 94305; ^d^Department of Psychology, New York University, New York, NY 10003; ^e^Department of Linguistics, New York University, New York, NY 10003; ^f^Ecole Normale Superieure, Paris Sciences et Lettres (PSL), CNRS, Paris 75005, France; ^g^Meta AI, Paris 75002, France

**Keywords:** speech, hierarchy, language, machine learning, decoding

## Abstract

Speech comprehension involves transforming an acoustic waveform into meaning. To do so, the human brain generates a hierarchy of features that converts the sensory input into increasingly abstract language properties. However, little is known about how rapid incoming sequences of hierarchical features are continuously coordinated. Here, we propose that each language feature is supported by a dynamic neural code, which represents the sequence history of hierarchical features in parallel. To test this “hierarchical dynamic coding” (HDC) hypothesis, we use time-resolved decoding of brain activity to track the construction, maintenance, and update of a comprehensive hierarchy of language features spanning phonetic, word form, lexical–syntactic, syntactic, and semantic representations. For this, we recorded 21 native English participants with magnetoencephalography (MEG), while they listened to two hours of short stories in English. Our analyses reveal three main findings. First, the brain represents and simultaneously maintains a sequence of hierarchical features. Second, the duration of these representations depends on their level in the language hierarchy. Third, each representation is maintained by a dynamic neural code, which evolves at a speed commensurate with its corresponding linguistic level. This HDC preserves the maintenance of information over time while limiting destructive interference between successive features. Overall, HDC reveals how the human brain maintains and updates the continuously unfolding language hierarchy during natural speech comprehension, thereby anchoring linguistic theories to their biological implementations.

## Significance

To understand speech, the brain generates a hierarchy of neural representations, which map from sound to meaning. We recorded whole-brain activity while participants listened to audiobooks and modeled neural activity using time-resolved machine learning methods. Our analyses reveal that different neural patterns are activated over time to process each feature of the hierarchy, and abstract features have a slower spatial trajectory than sensory features. This spatiotemporally dynamic code enables a local history of inputs to be encoded in parallel, across a hierarchy from sound to meaning, also in parallel. This leads us to propose a dynamic model of cortical language processing: hierarchical dynamic coding (HDC).

How the human brain rapidly and robustly extracts meaning from acoustic signals during speech comprehension remains a fundamental question in neuroscience. At the level of neural representation, evidence suggests that the brain transforms the sensory input into a hierarchical set of language features, which span from speech sounds to meaning (1).

One body of work has studied the spatial localization of this feature hierarchy using functional MRI (fMRI). Phonetic (2, 3), syllabic, (4, 5) and lexical features (6–8) and associated syntactic structure (9–12) are represented in the temporal, parietal, and prefrontal cortices, with more abstract linguistic representations encoded in more distributed and higher-level activation patterns (13–15).

The dynamics of speech processing have been studied in a complementary body of work using electroencephalography (EEG). Auditory responses to onsets, offsets, and fluctuations in loudness are associated with the N100 component, which peaks at approximately 100 ms (16). Surprisal associated with phonological input—for example, if a phoneme violates a task-induced phonological expectation—is associated with amplitude modulations 250 to 300 ms (17–19). This is referred to as the Phonological Mapping Negativity (PMN). Lexical and lexico-semantic processing are robustly indexed by the N400 component (approximately 250 to 500 ms), which is sensitive to semantic context as well as word-form features such as frequency and neighborhood size (20–22). Frontal negativities in similar time windows have also been linked to lexical–syntactic access, including distinctions between closed- and open-class words (23, 24). Finally, syntactic complexity and various kinds of anomalies elicit broad posterior positive deflection ~600 ms after word presentation—the P600 (25–29), and a sustained negative-going potential may reflect long-distance syntactic and semantic dependency (30, 31, 32–35). Another body of work using scalp EEG, intracranial EEG, or magnetoencephalography (MEG) has also begun to study multiple features in parallel, finding simultaneous encoding of language properties (8, 36–40).

Due to the compositional nature of language structure—that is, phonemes combine to make syllables, which combine to make words and phrases—hierarchical processing entails integrating information over variable and nested timescales to resolve feature identity (41–43). For example, evidence from behavioral reaction time studies and eye-tracking visual world paradigms has shown that listeners maintain lower-order information of speech for multiple seconds in the future, using them to refine lexical, syntactic, and semantic interpretations (44–46).

The longevity of language feature encoding has the clear computational advantage of enabling the composition and resolution of higher-order structures (47). However, it is difficult to reconcile this algorithmic finding with the current dominant position that there exists a one-to-one correspondence between a given language feature and its neural representation in space and time. This account fails to explain how the brain maintains low-level elements long enough to integrate them into more complex units (6), while continuously updating each element to keep up with the continuously unfolding speech stream to appropriately process new incoming information (41, 48, 49).

These constraints theoretically apply across all levels of the hierarchy: from assembling phonemes into words, to assembling words into sentences. A new computational framework with an updated view of neural implementation is thus essential to account for how the cortex simultaneously maintains and updates each of the representations of language to build increasingly high-level representations.

**Fig. 1.**
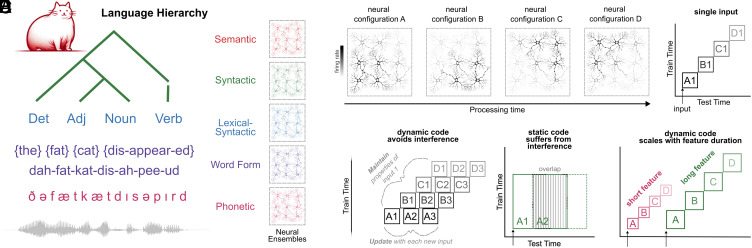
The hierarchical dynamic coding (HDC) hypothesis. (*A*) Schematic of the language hierarchy, from the acoustic input to the meaning of the utterance. Note that the image at the *Top* of the hierarchy is intended to represent the meaning of the entire sentence, not just the single word “cat”. Each feature of the hierarchy is hypothesized to be encoded by a distinct neural ensemble. (*B*) Schematic of the HDC hypothesis: for each feature of the hierarchy, encoding evolves across different neural ensembles as a function of time. (*C*) Schematic decoding result for a single speech input. Each neural configuration {A, B, C, D} is engaged in sequence, leading to a lack of generalization across all train/test times. (*D*) Schematic decoding result for sequences of inputs, which satisfies both the constraint to maintain information over time, and update as new inputs are received. This means that there is no representational overlap between neighbors in the sequence. (*E*) A static neural code, by contrast, implies a high degree of representational overlap between neighbors. (*F*) Schematic prediction that shorter and longer features of language will display distinct processing dynamics: Shorter features will evolve between neural codes faster and will be encoded for shorter duration; longer features will evolve slower and will be encoded for a longer duration.

Time-resolved decoding of brain activity may provide a promising tool to resolve this issue (50–52). By decoding the representations at each point in time, acoustic-phonetic (3) and visual features (53) have recently been shown to be embedded in a dynamic neural code. For example, in Gwilliams et al. (3), we provided evidence for a dynamic neural code that supports acoustic-phonetic processing in speech, but it remains fully unexplored whether a dynamic code underlies more abstract feature processing, and how the parameters of that code adjust as a function of hierarchical level (Fig. 1). We test whether this dynamic coding can be applied hierarchically to both maintain and update the many representations of language, while avoiding interference across successive phonemes, syllables, and words, henceforth referred to as hierarchical dynamic coding (HDC). We focus on representations at the level of words and sentences, rather than at the level of discourse.

While prior studies have shown that multiple linguistic features can be decoded from neural responses to speech (54), these efforts have largely focused on a limited set of dimensions of the linguistic hierarchy. For example, previous work compared acoustic and phoneme-level features such as the spectrogram, phonetic features, phonotactics, and phoneme surprisal and entropy, while others focused on the addition of the word-level feature word frequency, and lexical semantics (8, 38, 55, 56). Our study builds on this work by testing multiple dimensions of speech and language in a single study, across all hierarchical levels. This allows us to compare the strength, dynamics, and longevity of information encoding from phonemes to phrases, within a single, continuous speech paradigm.

We recorded MEG from 21 participants listening to two hours of audio stories. The data used here partially overlap with the data used in Gwilliams et al. (3) (see *Methods* for details). All participants were native English speakers, and the audiobooks were presented in English. We fit linear models (50) to decode 54 linguistic features organized into six levels of representation: phonetic, word form, lexical–syntactic, syntactic operation, syntactic state, and semantic. We address three main questions: i) Can we simultaneously decode all six levels of representation in the language hierarchy during continuous speech processing? ii) What are the relative onsets and durations of these hierarchical levels? and iii) Does their underlying neural code evolve over time, with speed commensurate to their level in the hierarchy (Fig. 1)?

## 1. Results

### 1.1. Robust Decoding of Speech Features.

**Fig. 2.**
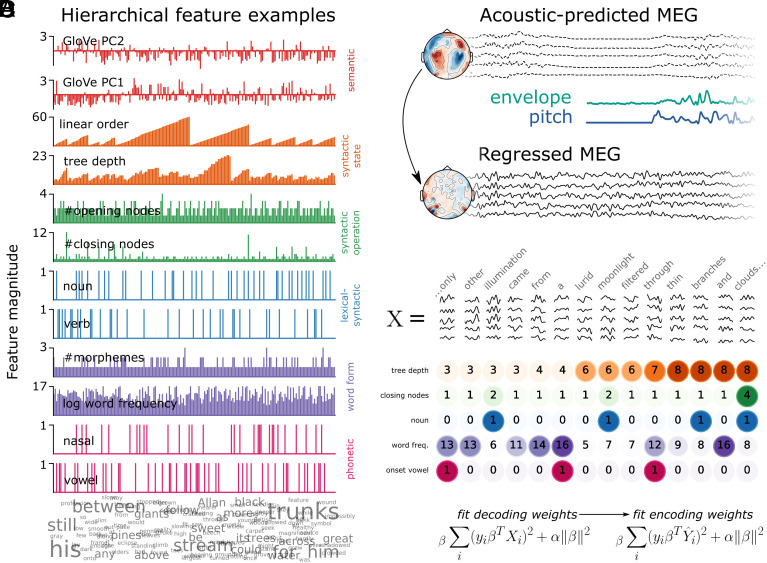
Methods. (*A*) Feature values are plotted for a selection of 500 consecutive words, selecting two example features for each level (from a total of 54 features). The level is displayed on the *Right*-hand side. Color corresponds to the language level it operationalizes. The word cloud at the *Bottom* displays each of the 500 words, adjusting the font size to be proportional to the number of instances in the story snippet. Note that the GloVe vectors are computed using a symmetric context window of size 10, and therefore they capture meaning beyond the single lexical item. (*B*) First, we fit a receptive field model based on the envelope and pitch of the acoustic speech input, and regress this out of the continuous MEG signal. The topography above shows sensor weights sensitive to acoustic features; the topography below confirms that sensor weights sensitive to acoustic features are at zero after this procedure. The time courses above correspond to the TRF predictions from the acoustic model; the teal and purple time courses correspond to the time course of the pitch values and envelope values; the bottom time course corresponds to the residual MEG data after the acoustic predictions have been subtracted out. (*C*) Data structure. The epoch data matrix *X* has shape words (8,000) × sensors (208) × time (201). Here, a schematic of the epochs is shown for a subset of the story. Below, a sample of 5 example features are displayed for each word. The superimposed number and color intensity correspond to the feature value at a particular word. (*D*) Main equations for the back-to-back regression method. α = the fitted regularization parameter. β = the fitted model coefficients of interest. First, we fit ridge regression to decode each feature *y* from the MEG signal *X*, then we evaluate the prediction of the features ($\hat{Y}$) against the true value of *y* with an encoding model.

**Fig. 3.**
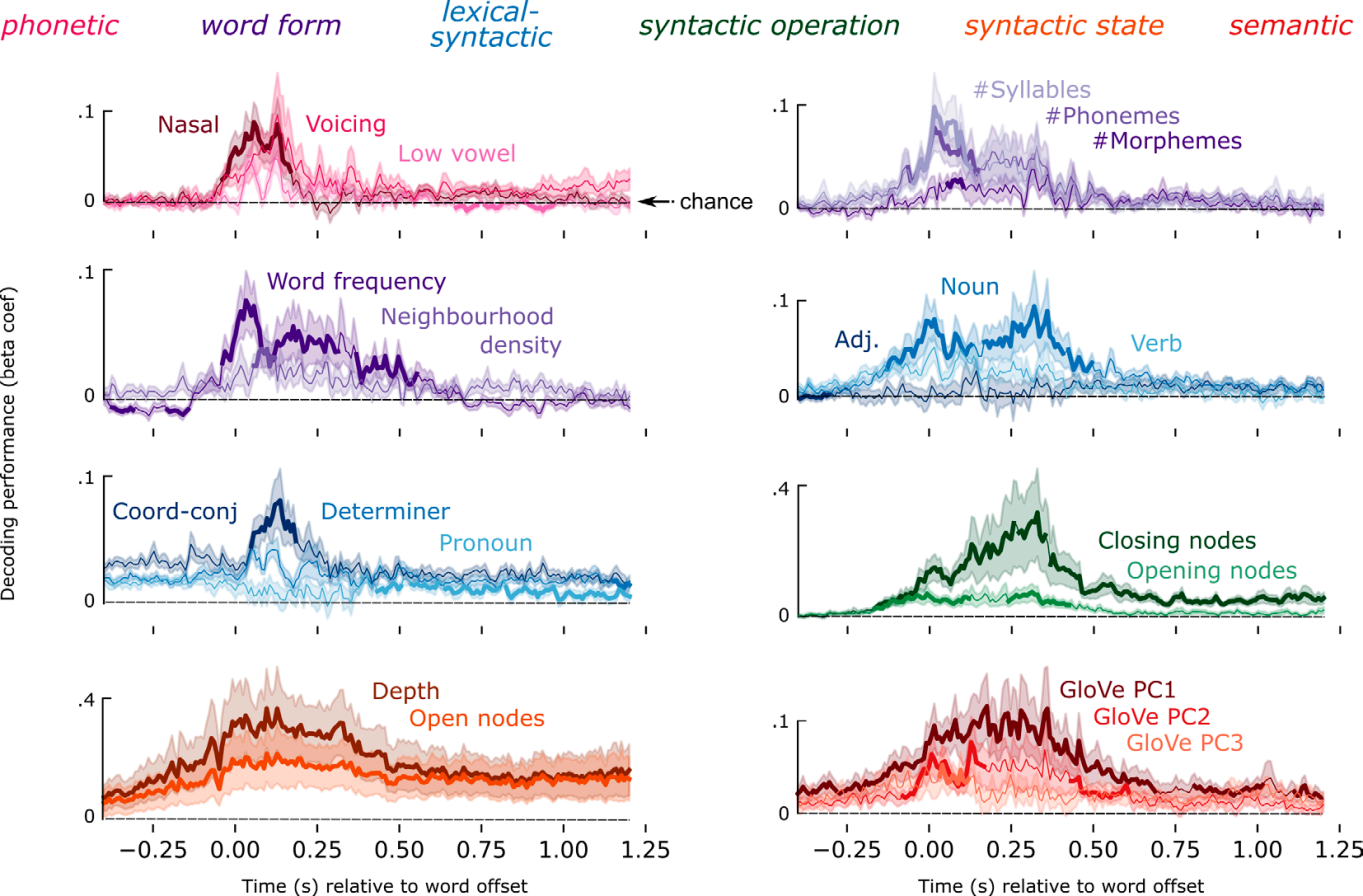
Feature decoding time courses. Time courses of decoding performance for a subset of language properties, locked to word offset. Line color corresponds to family assignment, which are listed above. Solid trace corresponds to mean performance across subjects; shading is the SEM across subjects. A bold mean trace corresponds to the result of a temporal permutation cluster test, indicating when the feature is decodable significantly better than chance. The dashed black line corresponds to chance-level performance.

Our first question is whether the rich suite of linguistic features can be simultaneously decoded from MEG activity during continuous listening. To evaluate this, we compute the time course of each linguistic feature (Fig. 2) and evaluate statistical significance with a temporal permutation cluster test of the distribution of beta coefficients across participants. Overall, our results show that we can precisely track a remarkably diverse set of linguistic features from MEG activity (Fig. 3). The results of the full statistical analyses on all features are provided in *SI Appendix*, Tables S1–S12. For the analysis on raw Spearman R correlation rather than B2B regression, see *SI Appendix*.

In all results that follow, we time-lock our analyses to word offset rather than word onset, because it provides significantly higher decoding performance in all of our analyses. If the reader is interested in the word onset-locked results, or the comparison between the two, please refer to *SI Appendix*, Supplementary Results.

**Fig. 4.**
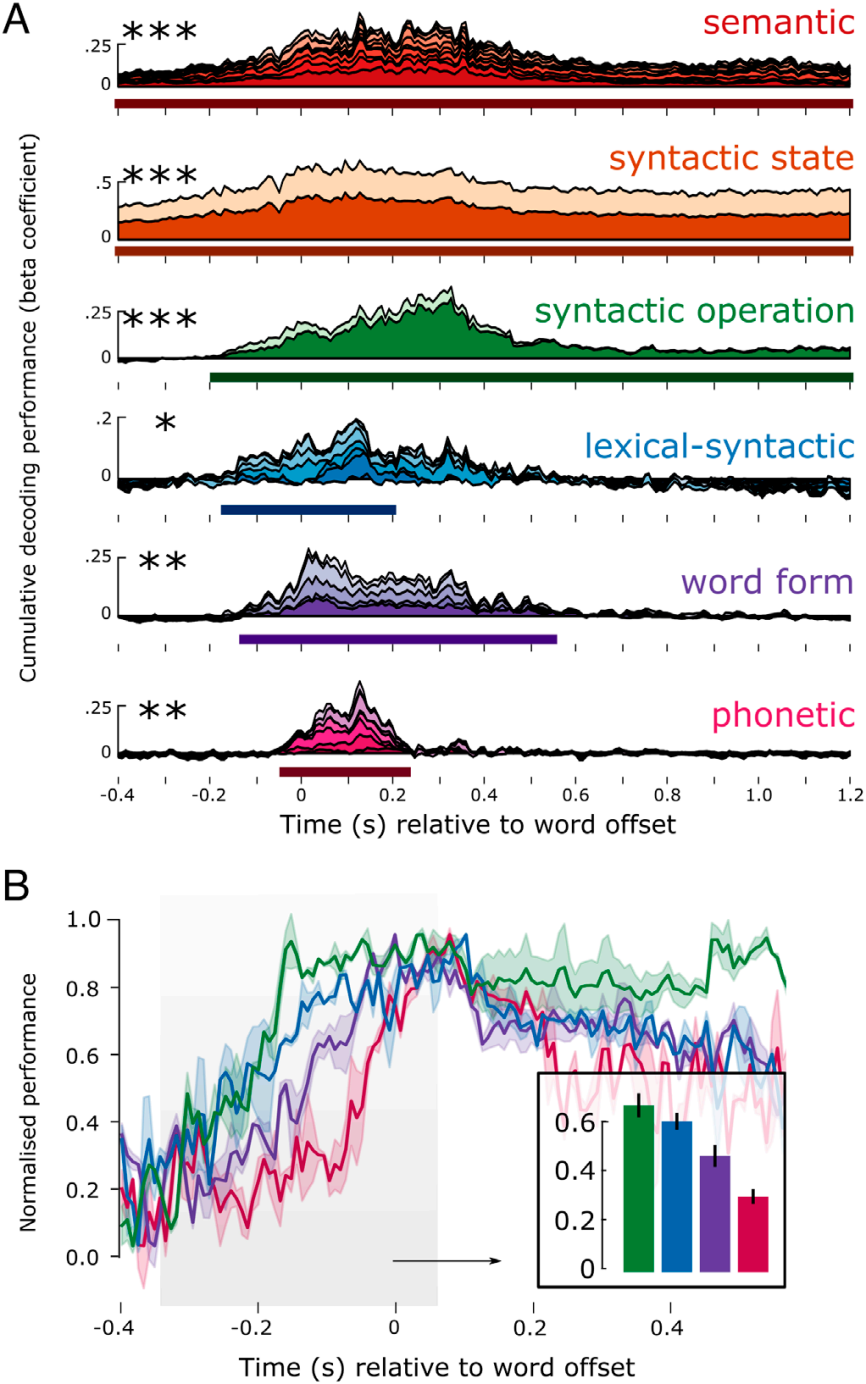
Decoding hierarchical features. (*A*) Result of decoding each language level over time. The beta coefficients of each feature are stacked on top of each other, such that the top of the time course plot corresponds to the cumulative sum of all features in that linguistic level. The x-axis corresponds to time in seconds relative to word offset. The y-axis corresponds to the cumulative beta-coefficient across features. The solid line below the time-course represents the extent of the significant temporal cluster; asterisks represent its significance. (*B*) Decoding performance zooming in for the lowest four feature families, and showing the SEM across subjects. Higher-level features come online earlier than lower-level ones. This is shown in the barplot, averaging performance before word offset shows a linear decrease in amplitude. *P < 0.05; **P < 0.01; ***P < 0.001.

To summarize the levels of the hierarchy that are robustly encoded in neural activity, we grouped decoding performance of the original 54 linguistic features into 6 feature families, and took the average of the features in a family, to plot 6 decoding time-courses (Fig. 4*A*). Features across all six feature families could be detected from MEG responses, with notable differences in latency and duration: On average, phonetic features were detectable from −40:230 ms [*ť* (average *t*-value in the cluster) = 2.57, *P* = 0.013] relative to word offset; word form features from −130:550 ms ($\hat{t}$ = 2.6, *P* = 0.002); lexical–syntactic from −170:200 ms ($\hat{t}$ = 2.18, *P* = 0.029); syntactic operation from −190:1,200 ms ($\hat{t}$ = 2.7, *P* < 0.001); syntactic state ($\hat{t}$ = 3.54, *P* < 0.001) and 20-word semantic field word embeddings ($\hat{t}$ = 3.46, *P* < 0.001) throughout the entire search window (Fig. 4*A*). Note that the GloVe vectors are trained using a symmetric context window of size 10, and therefore, they capture meaning beyond the single lexical item. This means that these features should be interpreted differently from a more local semantic feature like animacy or concreteness. These results are consistent across the two recording sessions (*SI Appendix*, Fig. S6), thus demonstrating internal replicability (see *SI Appendix* for detailed results). Overall, this analysis confirms that, during continuous speech listening, the brain builds a rich set of hierarchical linguistically motivated features.

### 1.2. The Timing of Linguistic Representations Depends on Their Level in the Language Hierarchy.

How do the latency and duration of each feature relate to their respective level in the linguistic hierarchy? To address this issue, we analyzed the average time-course of each of the 6 feature families (Fig. 4).

First, we assessed the relationship between hierarchy and decoding onset time. For this, we normalized the decoding performance for each feature family, by dividing the group average by its maximum, for each family separately. We analyzed the rise-time before word offset (for the analysis on word onset, see *SI Appendix*, Supplementary Figures). As shown in Fig. 4*B*, higher-level features were detectable earlier than lower-level features, resulting in a significant negative correlation between hierarchical level and the peak of the normalized performance (*r* = −0.82, *P* < 0.001).

Second, we tested the relationship between hierarchy and decoding duration. We found that higher-level features were decodable significantly longer than lower-level features, resulting in a significant positive correlation between level and duration (*r* = +0.75, *P* < 0.001). This effect was particularly striking for the syntactic and semantic features, which were decodable for over 1 s after word offset, continuing well into the processing of the subsequent words (see *SI Appendix*, Fig. S1 for the distribution of latencies of upcoming words). This systematic relationship between the longevity of encoding and the position of the level in the hierarchy also suggests that longevity is not trivially caused by a data preprocessing step, such as filtering, or inherent autocorrelation of the recording modality.

Third, we tested the extent to which different features of the hierarchy are represented in parallel. We found evidence for a nested temporal structure, whereby the decodable window of a given level (L) was generally contained within the decodable window of the feature at L + 1. For example, the start and end of significant phonetic decoding falls within the start and end of word form decoding, and that in turn within the start and end of lexical–syntactic decoding, etc. A one-way F-test revealed that the entire hierarchy as defined by the 6 feature families was decodable in parallel from −40:230 ms (*F*-value in the cluster = 4.1, *P* < 0.001) relative to word offset, i.e., throughout the duration of phonetic processing of the final speech sound of the word. Because the analysis is locked to the offset of the words in this analysis, we remove the confound that increasing language levels are instantiated in acoustic events that are, on average, longer in duration.

Together, these results confirm a key prediction of HDC: The dynamics of processing are increasingly sustained as the feature under consideration is high in the language hierarchy. We also observe that information at each level is encoded well into the processing of subsequent phonemes and words, leading to significant parallel processing, across and within levels of representation.

### 1.3. Hierarchical Features are Encoded in a Dynamic Neural Code.

We find that each linguistic feature can be decoded—and is thus represented—for a longer time window than its actual duration in natural speech. Here, we test the HDC hypothesis: A dynamic neural code allows successive phonemes, syllables, and words to be maintained without representational overlap between sequential neighbors.

To test this, we implemented a temporal generalization analysis (3, 50) (Fig. 1). This method involves evaluating whether the topographic pattern learned at time *t* generalizes to subsequent and preceding time-points (see *Methods* for details). If the representation is held within the same neural pattern over time, then the topographic pattern learned at time *t* should generalize to time *t* + N, leading to a “square” decoding matrix. By contrast, if the neural code evolves as a function of time, then the topographic pattern learned at time *t* would not be the same at time *t* + N, even if the representation can also be decoded at t + N. In this scenario of a dynamic code, we thus expect to detect a “diagonal” matrix.

Of primary interest are two parameters of this generalization matrix: (1) the window during which the representation can be decoded and (2) the window during which decoders tend to generalize.

**Fig. 5.**
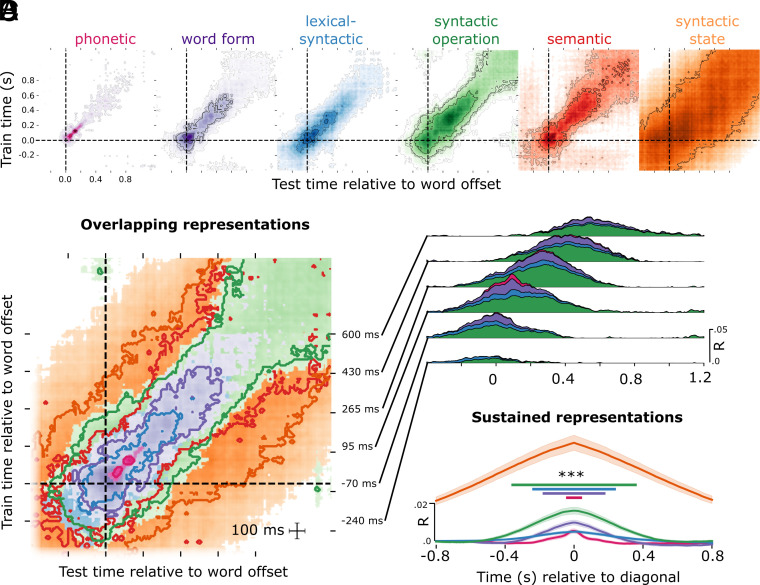
Evidence for HDC. (*A*) Temporal generalization analysis for each of the six linguistic levels of analysis. Each contour represents a significance threshold of *P* < 0.05, *P* < 0.01, *P* < 0.005, and *P* < 0.001. (*B*) Same data as shown in A but for just the *P* < 0.01 threshold. (*C*) Cumulative temporal generalization performance for the temporal decoders trained at different time-points relative to word offset, just for phonetic, word form, lexical–syntactic and syntactic operation. (*D*) Data realigned relative to the diagonal of the temporal generalization matrix, showing the relationship between format maintenance and feature complexity, here just for phonetic, word form, lexical–syntactic, syntactic operation, and syntactic state. ***P < 0.001.

We applied this analysis to each of our language features, and then averaged the generalization matrices over the six levels of interest (Fig. 5) to estimate the similarity of spatial evolution across the hierarchy.

We found that all six feature families are processed using a dynamic neural code. The neural activity patterns associated with each linguistic features are only stable for a relatively short time period: phonetic duration=184 ms; sustain = 64 ms; word form duration = 752 ms; sustain = 384 ms; lexical–syntactic duration = 536 ms; sustain = 224 ms; syntactic operation duration = 1392 ms; sustain = 720 ms; syntactic state duration = 1250 ms; sustain = 1600 ms). This means that all levels of representation across the hierarchy are supported by neural patterns that change over time.

Furthermore, the stability of a linguistic feature depends on its level in the language hierarchy: The lower-level phonetic features, which are defined over smaller linguistic units (phonemes), evolved significantly faster (average generalization time 64 ms), than lexical features (224 ms), and those, faster than syntactic features (730 ms). This led to a significant correlation between the location of the family in the hierarchy and duration of information sustain (*r* = −0.89, *P* = 0.034) (Fig. 5*D*). This finding suggests that while all levels of the hierarchy share a dynamic coding scheme, the speed with which information is routed to different neural patterns scales with unit duration and abstraction.

### 1.4. Simulating HDC.

Our results have revealed a number of potentially important components of the spatiotemporal dynamics of language encoding, and how they vary across the language hierarchy. In this final section, we perform several simulations to test the assumptions of the computational framework we are proposing.

**Fig. 6.**
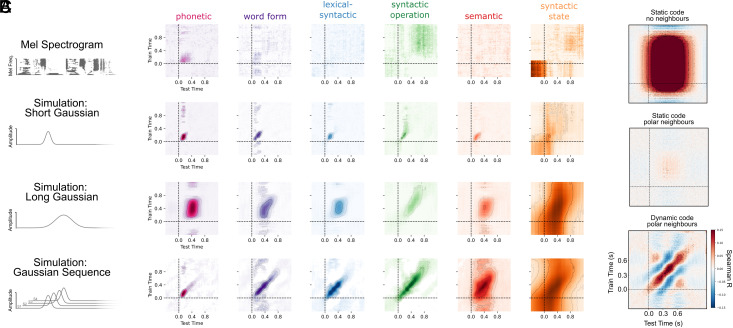
Simulating empirical results. (*A*) We performed four simulations: on the Mel spectrogram of the speech; simulating a short Gaussian response at linguistic feature onset; simulating a longer Gaussian response at linguistic feature onset; simulating a sequence of Gaussian responses, in line with the HDC hypothesis. (*B*) Results of temporal generalization analysis when applied to each of the simulated datasets. (*C*) Simulating static and dynamic neural codes, under conditions of sequence interference.

First, we repeated our hierarchical analyses on the Mel spectrogram of the speech signal to test to what degree the hierarchy of language features is linearly encoded in the acoustics that enter the ear. We computed the power in 50 log-spaced frequency bands of the spoken stories, spanning from 1 to 5000 Hz (*Methods*). From this spectral representation, we used Ridge regression to decode each of the 54 hierarchical language features described above, using the temporal generalization analyses described in Section 1.3. We find that phonetic and word form features can be decoded from the Mel spectrogram better than chance, as confirmed with a random-shuffle permutation test (*P* < 0.001). We also found that Syntactic Operation could be decoded late in the epoch time window, and Syntactic State could be decoded early in the epoch time window (both *P* < 0.001). Upon further inspection, we identified that this is caused by systematic co-occurrence with onsets from silence and offsets into silence (*SI Appendix*, Fig. S10). Lexical–syntactic features and the word embeddings were not decodable from the Mel spectrogram at any latency in the epoch. Together, this suggests that i) lower-level properties of speech are indeed linearly encoded in the acoustic input; ii) seemingly higher-order syntactic features have acoustic correlates, linked to the beginnings and ends of sentences; iii) lexical–syntactic and semantic features are not robustly encoded in the input (see Fig. 6 *B*, *Top* row and *SI Appendix*, Fig. S9). Overall, this supports that our results are not a trivial reflection of the input, but rather reflect the outcome of an active neural process applied to that input.

Second, we tested whether the dynamics we observe in Figs. 4*A* and 5*A* could be a consequence of the dynamics of the language features in sparsity or autocorrelation. For example, language properties at the “top” of the hierarchy such as syntactic depth have a higher autocorrelation, given that the value of depth at the current word is likely to be correlated with subsequent and preceding words. We simulated MEG responses to the features in our stories, preserving all native feature dynamics. Each feature was treated as a discrete impulse at word onset, which was convolved with a Gaussian kernel to generate a simple, time-limited neural response. The simulated response to each feature was summed across features. Please see *SI Appendix*, section 3.12 for more details.

We performed three simulations: i) spatially static short Gaussian response; ii) spatially static long Gaussian response; and iii) spatially dynamic sequence of Gaussian responses across sensors. For i), we simulated each Gaussian by selecting a peak time uniformly sampled from 100 to 200 ms; an amplitude uniformly sampled from −1 to +1, and a width uniformly sampled from 20 to 50 ms. For ii), we simulated each Gaussian by selecting a peak time uniformly sampled from 350 to 500 ms; an amplitude uniformly sampled from −1 to +1, and a width uniformly sampled from 100 to 150 ms. For iii), we used the Gaussian parameters from i) but additionally added a cascade of Gaussian responses, moving to distinct sensors each time. For each level of the hierarchy, we added an additional Gaussian response to the sequence, each spaced 50 ms apart, thus simulating the hypothesized neural code of HDC as outlined in Fig. 1.

Our simulations i) and ii) reveal that for all features other than Syntactic State, the onset and duration of feature encoding directly reflected the onset and duration of the ground-truth neural response (e.g., see *SI Appendix*, Figs. S11 and S12), and critically did not scale with the position of the level in the hierarchy. This means that the dynamics we observe empirically from our MEG decoding are crucially not merely a reflection of the dynamics of the stimulus features, but are the consequence of the spatiotemporal dynamics of the neural code applied to those features. In addition, the comparison between the “short” and “long” Gaussians allows us to test and confirm that additional smearing of MEG responses does not lead to the dynamic code we empirically observe. In simulation iii), we recapitulate our finding that a Hierarchical Dynamic Code gives rise to increased duration of encoding across each level of the hierarchy. We also find that only under the HDC simulation do we observe a “diagonal” generalization pattern, again providing evidence that the dynamics are not merely a consequence of the input. Finally, we also see that none of our simulations reveal a hierarchically driven adjustment of decoding longevity (*SI Appendix*, Figs. S9–S12), suggesting that the variation in acoustic duration across levels does not cause the longevity of neural encoding in our results.

The one exception to the above is the simulation of Syntactic State. We found that the duration of syntactic state encoded was much longer lived than the ground truth response vector. We attribute this to the extreme autocorrelation of this feature across word sequences. Consequently, we interpret the dynamics of this feature with caution, given that the prolonged encoding can also be recovered from a ground-truth transient Gaussian response. As noted, Syntactic State is the only feature with this property.

### 1.5. Simulating Destructive Interference.

That the neural code for shorter units evolved faster, and the neural code for longer units evolved slower, has the consequence that neighboring units avoid representational overlap across the linguistic hierarchy. In this final analysis, we test whether this serves to avoid “destructive interference” whereby the features of neighboring words would serve to cancel each other out, if the neural encoding of those words was shared. We define “interference” specifically as the case where two or more features of the input are encoded in neural activity at the same time, but their feature values are contrastive, thus leading to cancellation.

Here, we test our hypothesis that a static and prolonged neural code would lead to catastrophic interference between neighboring features. We simulated MEG responses as Gaussian activation functions, with a peak response at 400 ms, amplitude of 1.5 femto-tesla, and response width equal to the average word duration in our stories (293 ms). We simulated responses of this static code using i) exaggerated distance of 2 silent seconds between neighboring words; ii) actual distance between words from our story stimuli. To model maximal interference, we used a simulated feature vector that fluctuated between +1 and −1 at the onset of each word in the story. Finally, to simulate responses under the HDC hypothesis, we encoded the maximally contrastive simulated feature in a sequence of Gaussian responses that travel across space. Each Gaussian in the sequence had a peak response at 400 ms, amplitude of 1.5 femto-tesla, and response width equal to the average word duration in our stories (293 ms). There were 3 Gaussians in the sequence, occurring at 0 ms, 50 ms, and 100 ms relative to feature onset.

We determined whether representational interference had occurred by decoding the ground-truth simulated vector back from each of the three simulated MEG responses. We applied temporal generalization analyses to our simulated MEG responses, and we found that when the silent spaces between neighboring words were sufficiently exaggerated, the simulated feature vector could be accurately reconstructed. By contrast, when we used the actual rapid pace of words in our stories, as is representative of real speech, we found evidence for catastrophic interference, and the underlying feature vector could no longer be recovered. Finally, when the feature was encoded in a dynamic code that processed across space, the ground-truth contrastive vector was again recoverable (Fig. 6*C*).

## 2. Discussion

Speech comprehension hinges on a delicate balance between *maintaining* low-level elements—like words, long enough to integrate them into more complex units—like phrases (57), while continuously *updating* each element in time with rapidly incoming information (41, 48, 49). The neural implementation of this process must enable multiple features across the hierarchy, across an extended history, to be encoded simultaneously, while avoiding destructive interference between neighboring inputs (58).

We propose a processing model that satisfies these constraints: HDC. Our computational account, and associated neural description, challenges models that posit a one-to-one mapping between a neural pattern and language feature (13, 14). Rather than a singular response latency (26, 59) at a given spatial location (9, 60), we find that language representations are encoded in a series of spatial patterns over time, and the full hierarchy is encoded largely in parallel. This provides an updated neuroscientific framework for interpreting the substantial body of behavioral research that demonstrates longevity of hierarchical representations (44–46).

Our results also extend prior decoding studies by incorporating syntactic dimensions into the hierarchical analysis being conducted. While previous multivariate studies have focused on lower-level dimensions such as phonetic or lexical identity, the simultaneous inclusion of syntactic operations—such as node closure—provides a rich view of how abstract structural information is encoded and maintained during language processing. This expansion in representational scope enables comparisons across linguistic levels and supports a more complete account of hierarchical processing dynamics in naturalistic language comprehension.

Because the dynamic neural code systematically traverses neural patterns over time, each pattern is effectively “time-stamped” according to its absolute elapsed time since the feature began. We observe that this dynamic code evolves at different speeds across the hierarchy: Higher-level representations shift between patterns more slowly than lower-level ones. This means lower-level representations have a finer-grained temporal code — since neural patterns closer in time are more distinct — while higher-level representations span broader time intervals. These distinct time-stamping timescales resemble the hierarchical sinusoidal positional embeddings used in transformer models (61). And the use of distinct subspaces to encode relative position is similar to the rotary position embedding (RoPE) used in speech and language models (62, 63), whereby a continuously evolving projection helps the model to temporally locate, for example, the relative position of each word in a sentence, without the need for explicit ordinal position encoding (64). While we do not leverage transformer models in the present study, it would be interesting to explicitly evaluate the similarity between the RoPE mechanism and the evolving dynamic code we report as part of HDC. It would also be interesting to assess the geometric properties of the subspaces being traversed—for example, to test whether they are orthogonal.

In addition, higher-level features are maintained for significantly longer in the neural signal, extending into the processing of multiple words in the future. This neural overlap is much greater than previously appreciated (2, 9). It is possible that the information remains available to the system as “resonance” encoding, but does not reflect an active ongoing cognitive process—this is not something our decoding analysis can disentangle. However, we speculate that this sustained encoding provides three key processing advantages. First, it allows the system to build sufficiently long sequences to construct higher-level representations when operating in a bottom–up manner. Second, it enables bidirectional interaction between features in both a top–down and bottom–up direction. Third, it supports the formation and integration of predictions both within and across levels of representation.

In addition to sustained feature encoding, we observe significant anticipatory encoding before the word is available in the input. This could be an indication of the brain’s *prediction* about the upcoming feature, which is later evaluated relative to the actual feature outcome—leading to the surprisal response (65, 66). This is in line with recent EEG studies investigating “preactivation” of expected feature outcomes, using highly constraining sentences (67–69). This is also in line with the idea that higher-level representations can maintain stable states over extended timescales and feed top–down constraints down to lower-level processing—a hallmark of hierarchical predictive coding models (36, 66, 70).

This top–down process is consistent with the “Good Enough” and “Syntax First” models of language processing (71–74), where higher-order structures are used to guide comprehension based on what is coherent in context. This “reverse” order of speech processing presents three computational advantages (75, 76). First, because higher-level language features are abstracted away from the sensory signal, comprehension is more robust to auditory noise and ambiguity (77, 78). Second, ambiguity at one level of representation may be resolvable by integrating information from other levels (79–82). Finally, it potentially speeds up processing by initiating high-level computations early during comprehension, rather than waiting for them to be formed compositionally in purely bottom–up fashion (71, 72, 74).

This account forms the hypothesis that when the listener is hearing the beginning of the story, the onset of feature encoding must proceed in the more traditional bottom–up order, given that there is minimal context to leverage for predictions. As the story unfolds, the brain can increasingly form predictions about upcoming inputs, thus leading to increasingly earlier feature encoding. Future studies should test whether this account bears out—that the direction and steepness of onset decoding directly scale with the predictability of upcoming inputs.

Our simulation analyses confirm that the dynamical speed of evolution, and the duration of encoding, are not trivial consequences of the dynamics of the features themselves, but rather are the result of an active process that the brain applies to these neural representations. Anatomically, these results align with the finding that higher-level areas (e.g. fronto-parietal cortices) integrate speech representations over longer time periods than lower-level areas (e.g., Heschl’s gyrus) (36, 43, 83–85).

Notably, the two syntactic levels—syntactic state and syntactic operation—revealed significantly different dynamics. The syntactic operation level, which includes features such as number of opening and closing nodes, evolved more quickly and was sustained for less time than syntactic state. Our simulation analyses help to clarify this difference: Even when we only simulate a single Gaussian response to the syntactic state feature (Fig. 6), the decoding dynamics out-live the ground truth neural generators. This is due to the extremely high autocorrelation of the syntactic state feature as compared to the other features. In addition, syntactic state was significantly decodable from the Mel spectrogram, which we show is due to covariance with sentence offsets. As a consequence, our acoustic control analysis and simulation results show that the dynamics of syntactic state are partially driven by the autocorrelation of the features at that level, and should be interpreted with caution. None of the other feature dynamics were explainable in this way—suggesting that the dynamics at all other hierarchical levels are indeed due to dynamics of the given neural process.

A major computational advantage of adjusting evolution speed with hierarchical level is the avoidance of destructive interference between neighboring elements of the speech input. If two or more features of the input are encoded in the same neural activity pattern, and their feature values are contrastive, we show in simulation that this prevents robust recovery of the underlying sequence representation. Given that phoneme sequences occur in more rapid succession than word or phrasal sequences, it is necessary for the dynamic code of phonemes to evolve more quickly to avoid representational overlap. When we simulate speech with exaggerated silences between features, static code destructive interference is resolved; however, the speech slow-down is suboptimal from an efficient communication standpoint (86), and would require information to be maintained for significantly longer periods. Thus, HDC provides an explicit account of how this dynamic code allows speech to unfold rapidly while serving to avoid destructive interference between neighboring inputs.

We observe that all levels of linguistic encoding, from sound to meaning, are supported by a dynamic neural code. This raises the question of whether this dynamic code may be observable for other sequential stimuli and in other sensory modalities. Previous work that focuses on just one level of representation has shown that novel visual stimuli, either presented in isolation (87) or presented in quick succession (53) as well as nonspeech frequency-modulated tones (88) also elicit a dynamic “cascade” of activity. This suggests that the dynamic code is not only recruited for highly trained stimuli, or only for auditory stimuli, but rather this is a “canonical” process that is applied across multiple processing domains. Previous work (88) associated different points in the response to auditory tones with sensitivity to different aspects of the experimental design: sensory ambiguity and contextual prediction. This suggests that the information that is encoded in the evolving neural pattern may also be changing over time—but, all “versions” of information are sufficiently correlated with our feature probes to provide successful readout throughout the response time course. Key questions to address in future work are whether, and in what ways, the information at each hierarchical level is changing over time. In addition, intracranial data would provide precise insight into *how* the spatial pattern evolves: How “far” does the neural code move; is it primarily within a brain region, or does information move across brain regions? Answers to both questions would help build a detailed understanding of the formats of information that are available to downstream areas at different latencies in processing, as well as the neural computations in place that modify the representational format through this processing pipeline.

Previous research investigating the dynamics of language feature processing using scalp EEG has studied many of the same levels of representation we explore here, by manipulating the expectation of one hierarchical dimension at a time, and observing corresponding temporal fluctuations in activity strength (21). Studies report that expectancy of lower-order phonetic features (38) leads to responses earlier than manipulations of predictably higher levels, including semantics and syntax (27, 28, 89, 90). Our findings contrast with these prior results, showing that the onset of higher-level feature encoding actually precedes the onset of lower feature encoding. We reconcile these findings by noting an important difference in our approach: We are decoding the *value* of the feature directly (e.g., “Noun”) rather than the surprisal of that feature (e.g., log probability of Noun). Studies exploring EEG responses during continuous speech have found that surprisal across the hierarchy is aligned with more traditional EEG studies—that is, surprisal of phonetics precedes surprisal of semantic and syntax—and they do not observe the “reverse” hierarchical encoding pattern we report here. Another difference is that we locked our analysis to word offset rather than word onset, because it consistently yielded more robust decoding performance; however, when we lock our analysis to word onset, we find a qualitatively similar pattern. This suggests that it is our choice of feature set, rather than our use of a naturalistic paradigm, or choice of event-locking, that explains the difference in dynamics. Finally, in using multivariate analyses, we are able to capture information that is encoded in patterns of activity that only weakly covary with signal strength at a single sensor, in addition to the high SNR signal changes that primarily drive univariate ERP-style responses. This may also contribute to our ability to detect low-amplitude activity patterns that may be invisible to ERP analyses.

We note that while we observed distinct dynamics across the hierarchy, the time course of decoding for individual features within a given level was somewhat heterogeneous (Fig. 3), in terms of decoding performance and time course morphology. Of relevance is that the six levels of representation selected here are not of a “natural kind”, but rather represent a theoretically driven discretization of a representational continuum from more sensory to more symbolic features. Precisely how this feature space gets carved into representational levels, and the choice of which features are averaged to create those levels, will likely affect the specific dynamics observed. Thus, we interpret our results as revealing hierarchical processing with flexible dynamics and overlapping gradients, rather than hard, fixed stages of processing.

Overall, our results offer an updated computational account, and associated neural description, of how the brain maintains and updates the continuously unfolding hierarchical representations of spoken language. We track a comprehensive hierarchy of speech and language dimensions, ranging from phonemes to syntactic trees, and find evidence for a canonical dynamic code, which adapts its processing speed as a function of level in the language hierarchy. HDC elegantly balances the preservation of information over time with minimizing neural overlap between consecutive language elements. This system provides a clear view of how the brain may organize and interpret rapidly unfolding speech in real time, linking linguistic theories with their neurological foundations.

## 3. Methods

We note that we are analyzing a naturalistic dataset of participants listening to short stories, which was also analyzed in a previous study from these authors: Gwilliams et al. (3). In the previous study, we focused our analysis purely on the acoustic and phonetic levels of processing and analyzed the responses to 50,518 phonemes per participant. Here, we are focusing our analyses on the 13,798 words and analyzing responses relative to a comprehensive *hierarchy* of language representation—significantly moving beyond the level of acoustic-phonetics. We note that all of the data preprocessing steps are the same as described in our prior paper. But the features we explore, the analyses we apply, and the simulations we run, are unique to the current paper.

For a full and detailed description of *Methods*, please refer to the *SI Appendix*.

## Supplementary Material

Appendix 01 (PDF)

## Data Availability

Raw data, annotations and preprocessed data have been publicly released on the Open Science Framework [https://osf.io/ag3kj/, (91)].
