# Supplementary material for: Hierarchical dynamic coding coordinates speech comprehension in the human brain
Source: Proc Natl Acad Sci U S A. 2025 Oct 17;122(42):e2422097122. doi: 10.1073/pnas.2422097122 (PMC12557530; doi:10.1073/pnas.2422097122)
Supplement: Supplementary file 1 — Appendix 01 (PDF) [file pnas.2422097122.sapp.pdf]

Hierarchical dynamic coding coordinates speech comprehension in the human brain

Supplementary Methods

Laura Gwilliams[1, 6, 7], Alec Marantz[2, 5] David Poeppel[2] & Jean-Remi King[3, 4]

[1]Department of Psychology, Stanford University

[2]Department of Psychology, New York University,

[3]Ecole Normale Superieure, PSL, CNRS,

[4] Meta AI,

[5]Department of Linguistics, New York University,

[6]Wu Tsai Neurosciences Institute, Stanford University

[7]Stanford Data Science, Stanford University

Supplementary Methods

We would like to note that we are analysing a naturalistic dataset of participants listening to short stories, which was also analysed in a previous study from these authors: Gwilliams et al., 2022, *Nature Communications*. In the previous study, we focused our analysis purely on the acoustic and phonetic levels of processing, and analysed the responses to 50,518 phonemes per participant. Here, we are focusing our analyses on the 13,798 words and analysing responses relative to a comprehensive *hierarchy* of language representation–significantly moving beyond the level of acoustic-phonetics. We note that all of the data preprocessing steps we outline below are the same as described in our prior paper. But the features we explore, the analyses we apply, and the simulations we run, are unique to the current paper.

1.0. Definition of terms:

- **Feature**: a property of language: e.g., “fricative” phonetic feature; word frequency, etc.
- **Level**: a group of features at the same degree of hierarchical position: e.g. phonetic, word form, lexical-syntactic, syntactic operation, semantic, syntactic state
- **Representation**: neural encoding scheme of a feature
- **Dynamic coding**: when neural representations are instantiated by distinct neural patterns over time.
- **Hierarchical Dynamic Coding**: when different levels follow a dynamic coding scheme

1.1. Participants

Twenty-one native English participants were recruited from the NYU Abu Dhabi community (13 female; age: M=24.8, SD=6.4). All provided their informed consent and were compensated for their time. Participants reported having normal hearing and no history of neurological disorders. Each subject participated in the experiment twice. Time between sessions ranged from 1 day to 2 months. All participants gave their informed consent, and the experiment was approved by the local IRB committee of NYU Abu Dhabi.

1.2. Stimulus development

Four fictional stories were selected from the Open American National Corpus [1]: Cable spool boy (about two bothers playing in the woods); LW1 (sci-fi story about an alien spaceship trying to find home); Black willow (about an author struggling with writer’s block); Easy money (about two old friends using magic to make money).

Stimuli were annotated for phoneme boundaries and labels using the ‘gentle aligner’ from the Python module *lowerquality*. Some prior testing provided better results than the Penn Forced Aligner [2]. To verify that the forced alignment did not have a systematic bias, we checked the MEG decoding of phonetic features for each sound file separately. In some cases, the aligner failed to find an alignment; we removed all such words from all analyses presented here.

Each of the stories were synthesised using the Mac OSX text-to-speech application. Three synthetic voices were used (Ava, Samantha, Allison). Voices changed every 5-20 sentences. The speech rate of the voices ranged from 145-205 words per minute, which also changed every 5-20 sentences. The silence between sentences randomly varied between 0-1000 ms.

1.3. Procedure

Before the experiment proper, the participant was exposed to 20 seconds of each speaker explaining the structure of the experiment. This was designed to help the participants attune to the synthetic voices.

The order of stories was fully crossed using a Latin-square design. Participants heard the stories in the same order during both the first and second sessions. This was in order to make direct comparisons between the first and second sessions.

Participants answered a two-choice question on the story content every ~3 minutes. For example, one of the questions was “what was the location of the bank that they robbed”? The purpose of the questions was to keep participants attentive and to have a formal measure of engagement. All participants performed this task at ceiling, with an accuracy of 98%. Participants responded with a button press. Stimuli were presented binaurally to participants though tube earphones (Aero Technologies), at a mean level of 70 dB SPL. The stories ranged from 8-25 minutes, with a total running time of ~1 hour.

The stimuli used for this study are detailed in Gwilliams et al., (2023) *Scientific Data*[3]. The stimuli of text, sound and timing are available on https://osf.io/ag3kj/.

Stories. Each participant listened to four fictional stories, over the course of two ~1h-long MEG sessions, with the exception of 5 subjects who only underwent 1 session. The stories were played in different orders across participants. These stories were originally selected because they had been annotated for their syntactic structures[1]. The corresponding text files can be found in stimuli/text/*.txt

Word lists and pseudo-words. To potentially investigate MEG responses to words independently of their narrative context, the text of these stories have been supplemented with word lists. It is common to use “word lists” as a baseline condition in labs using fMRI to study language processing (e.g., for a review see [4]. Specifically, a random word list consisting of the unique content words (nouns, proper nouns, verbs, adverbs and adjectives) selected from the preceding text segment was added in a random order. In addition, a small fraction (<1%) of non-words were inserted into the natural sentences of the stories. Again, the reason here is because non-words are also often used as a baseline comparison condition in neurolinguistics. The corresponding text files can be found in stimuli/text_with_wordlist/*.txt.

Importantly, the brain responses to these word lists and to these pseudo words are fully discarded from the present study. This had no negative bearing on the accuracy of the timestamps.

Given that we computed the syntactic features without the pseudo-word insertions, their inclusion in the stories likely did lead to weaker results than we would have observed had we not included these items in the stories.

Audio synthesis. Each of these stories was synthesized with Mac OS Mojave © version 10.14 text-to-speech. Voices (n=3 female) and speech rates (145 - 205 words per minute) varied every 5-20 sentences. The inter-sentence interval randomly varied between 0 and 1,000 ms. Both speech rate and inter-sentence intervals were sampled from a uniform distribution. Each `text_with_wordlist` files was divided into ~3 min sound files, which can be found in stimuli/audio/*.wav.

Forced Alignment. The timing of words and phonemes were inferred from the forced-alignment between the wav and text files, using the ‘gentle aligner’ from the Python module lowerquality (https://github.com/lowerquality/gentle). We discard, from subsequent analyses, the words that did not get a forced alignment through this procedure. Because the aligner uses a context window to determine alignment, there were some moments where uncertainty in the alignment of a single word resulted in full sentences being absent from the alignment[5]. Analysis of the Mel spectrogram and of the phonetic decoding led to better results when using gentle than when using the Penn Forced Aligner. The timing of each word and phoneme can be found in the events.tsv of each individual recording session.

Verification. To verify that the forced alignment did not introduce systematic bias, we considered several potential forms of misalignment. These included consistent global shifts in timing (where phoneme boundaries might be systematically too early or too late), systematic differences in alignment across phoneme types (such as nasals versus plosives), and position-related errors (such as worse alignment at the beginning of sentences, where the aligner might have less contextual information).

We evaluated these possibilities using two approaches. First, we manually reviewed approximately 10% of the aligned phonemes. A trained phonetician compared the time-aligned phoneme boundaries to the speech spectrogram in the software Praat and confirmed that the placements were generally accurate and did not show any consistent bias. Second, we examined the accuracy of MEG-based decoding of phonetic features across individual sound files. If there were systematic alignment errors, they would likely reduce decoding accuracy or introduce variability across recordings. However, we observed no such patterns, suggesting that the forced alignment was sufficiently precise and unbiased for use in our analyses.

We could not compute a formal measure of forced alignment accuracy due to the absence of ground-truth phoneme onset times in our dataset; however, prior work on comparable alignment systems, such as the NeuFA aligner and Kaldi-based tools, reports average phoneme alignment errors in the range of 15 to 25 milliseconds for clean speech, which provides a rough benchmark for the expected accuracy of Gentle under our recording conditions.

1.4. MEG acquisition

Marker coils were placed at the same five positions to localise each participant's skull relative to the sensors. These marker measurements were recorded just before and after the experiment in order to track the degree of movement during the recording.

MEG data were recorded continuously using a 208 channel axial gradiometer system (Kanazawa Institute of Technology, Kanazawa, Japan), with a sampling rate of 1000 Hz and applying an on- line low-pass filter of 200 Hz.

1.5. Preprocessing MEG

The raw MEG data were noise reduced using the Continuously Adjusted Least Squares Method (CALM: [6], with MEG160 software (Yokohawa Electric Corporation and Eagle Technology Corporation, Tokyo, Japan).

The data were bandpass-filtered between 0.1 and 50 Hz using MNE-Python's default parameters with firwin design [50] and downsampled to 250 Hz. We used MNE-Python version 1.3.0.

We segmented the data into epochs, from 400 ms pre-word onset to 1200 ms post-word onset. This modifies the data structure from being of [sensors x continuous_time] into [sensors x 1600ms x words]. No baseline correction was applied. This epoching step facilitates the decoding analysis method. Because we fit a Ridge regression decoding model at each lag separately relative to the boundary between words. Epoching the data relative to word offset (in the Main analysis) and relative to word onset (in the Supplementary analysis) makes the analysis procedure convenient to implement algorithmically, because we can fit the Ridge regression on each time lag in parallel.

1.6. Effects of acoustic features

In order to promote our decoding algorithm to identify neural patterns that correspond to language encoding per se, rather than low-level acoustic fluctuations in amplitude and pitch, we decided to regress out these features from the MEG data before applying our decoding method. When comparing the results to the non-regressed data, we found that this step did not make a difference to the interpretations of our results; however, given that it was a part of our initial analysis plan, we decided to keep this processing step to avoid introducing additional experimental degrees of freedom.

We used a temporal receptive field (TRF) model to regress from the raw MEG data responses that were sensitive to fluctuations in the pitch and envelope of the acoustic speech signal. We used the *ReceptiveField* function from MNE-Python [7], using ridge regression as the estimator and laplacian regularization. We tested ten lambda regularization parameters, log-spaced between 1^{-6} and 1^{+6}, and picked the model with the highest predictive performance averaged across sensors. MEG sensor activity

at each ms were modeled using the preceding 200 ms of envelope and pitch estimates. Both the acoustic and MEG signals were demeaned and scaled to have unit variance before fitting the model. MEG acoustic-based predictions were then transformed back into original MEG units before regressing out of the true MEG signals. This process, including fitting hyper-parameters, was applied for each story recording and for each subject separately, across 3 folds. This yields a de-confounded MEG dataset on which to continue our analysis.

1.7. Modeled features

We investigated whether single-trial sensor responses varied as a function of 54 features. Features spanned different levels of the linguistic hierarchy and included both binary and continuous variables.

1.7.1. Phonetic

Phonetic features were derived from the multi-value feature system reported in [8]. Note that this feature system is sparse relative to the full set of distinctive features that can be identified in English; however, it serves as a reasonable approximation of the phonemic inventory for our purposes.

Voicing. This refers to whether the vocal cords vibrate during production. For example, this is the difference between *b* versus *p* and *z* versus *s*.

Manner of articulation. Manner refers to the way by which air is allowed to pass through the articulators during production. Here we tested five manner features: fricative, nasal, plosive, approximant, and vowel.

Place of articulation. Place refers to where the articulators (teeth, tongue, lips) are positioned during production. For vowels, this consists of: central vowel, low vowel, mid vowel, high vowel. For consonants, this consists of: coronal, glottal, labial and velar.

Feature Category	Description	Feature Values	Examples
**Voicing**	Whether the vocal cords vibrate during production.	**Voiced** (vocal cords vibrate)	**Voiced**: b, d, g, z
		**Voiceless** (no vibration)	**Voiceless**: p, t, k, s
**Manner of Articulation**	How air passes through the articulators during speech.	**Fricative** (continuous airflow with friction)	**Fricative**: f, s, v, z
		**Nasal** (air passes through the nose)	**Nasal**: m, n
		**Plosive** (airflow is stopped, then released)	**Plosive**: p, t, b, d
		**Approximant** (articulators come close, but no friction)	**Approximant**: w, r, l, j
		**Vowel** (open vocal tract, no significant constriction)	**Vowel**: a, e, i, o, u
**Place of Articulation (Vowels)**	Where the tongue is positioned in the mouth.	**Central** (tongue in the middle)	**Central**: ə (schwa)
		**Low** (tongue positioned low)	**Low**: æ (as in *cat*)
		**Mid** (tongue positioned mid-height)	**Mid**: e (as in *bet*), o (as in *bore*)
		**High** (tongue positioned high)	**High**: i (as in *see*), u (as in *blue*)
**Place of Articulation (Consonants)**	Where the articulators (tongue, teeth, lips) shape the sound.	**Coronal** (tongue tip/blade)	**Coronal**: t, s, d, z
		**Glottal** (vocal cords)	**Glottal**: h
		**Labial** (lips)	**Labial**: p, b, m
		**Velar** (back of tongue)	**Velar**: k, g, ŋ (as in *sing*)

1.7.2. Word Form

To compute word form features, we used the English Lexicon Project (ELP) [9], which is a comprehensive database of English words that provides detailed phonological, morphological, and frequency-based metrics, which includes a corpus of spoken English. The ELP corpus includes a large collection of words from spoken and written English, allowing for a systematic quantification of various linguistic properties.

One key set of features includes the number of phonemes, syllables, and morphemes in each word. These measures provide a structural breakdown of the word into its constituent units, capturing its complexity at different linguistic levels. Phonemes represent the smallest units of sound that distinguish meaning, syllables structure the pronunciation and rhythm of a word, and morphemes reflect its smallest meaningful components, including roots and affixes. Words with more phonemes or syllables may be more challenging to articulate and process, while those with more morphemes may carry greater semantic complexity due to their morphological composition. These counts allow for a finer analysis of how word structure influences cognitive and linguistic processing.

A related measure is the number of phonemes within a syllable, which reflects the internal complexity of a word’s syllabic structure. Some syllables contain only a vowel (e.g., "a"), while others have multiple consonants clustered together (e.g., "strength"). This measure provides insight into the phonological

density of words, which can impact pronunciation difficulty and processing time in both spoken and written language.

In addition to structural features, we also considered word frequency, which was derived from the subtitles corpus of American English within the ELP. Rather than using raw frequency counts, we applied a log transformation to normalize the distribution, as word frequency follows a highly skewed pattern—some words appear extremely often (e.g., the, is), while others are much rarer (e.g., serendipity, quixotic). Word frequency is a crucial predictor in psycholinguistics, as high-frequency words tend to be recognized and retrieved more quickly and accurately compared to low-frequency words. This effect is well-documented in lexical decision tasks, reading studies, and spoken word recognition research.

Finally, we incorporated phonological neighborhood density, a measure of lexical competition. This metric captures how many other words can be formed by changing a single phoneme in the target word. For example, "cat" has many phonological neighbors (bat, mat, cut), making it part of a dense neighborhood, while "orange" has very few close phonological relatives and is in a sparse neighborhood. Words in dense phonological neighborhoods tend to experience more competition during retrieval, potentially slowing recognition and increasing processing difficulty. This effect has been widely studied in models of spoken word recognition and lexical access, as it influences both comprehension and speech production.

Together, these features provide a detailed linguistic profile of words, integrating phonological, morphological, and frequency-based dimensions. Their inclusion is essential for studying how different properties of words influence cognitive processing, whether in psycholinguistic experiments, computational models, or applied linguistic research.

1.7.3. Lexical-syntactic

Our lexical-syntactic level contains part of speech labels for every word. We derived these labels from the syntactic parse of the stories. This means that each word was assigned a syntactic category based on its role within the sentence structure, following standard linguistic parsing techniques.

To facilitate analysis, these word class labels were dummy coded relative to a set of 11 distinct word-class categories. Dummy coding is a common technique in statistical modeling and machine learning, where categorical variables are represented as binary indicators (0 or 1) for each possible category. This allows for flexible and interpretable numerical representation of categorical linguistic features.

The 11 word-class categories included in our coding scheme are:

- Adjective – Descriptive words that modify nouns (e.g., blue, tall).
- Coordinating conjunction – Words that link clauses, phrases, or words of equal grammatical rank (e.g., and, or).
- Determiner – Words that introduce and specify nouns (e.g., the, a).
- Noun – Words representing people, places, things, or ideas (e.g., house, girl).
- Pronoun – Words that replace nouns to avoid repetition (e.g., she, they).
- Preposition – Words that indicate relationships between elements in a sentence, often expressing direction, place, or time (e.g., under, on).
- Adverb – Words that modify verbs, adjectives, or other adverbs, often describing manner, time, or degree (e.g., slowly, fast).
- Verbal preposition – A specific type of preposition that functions as part of verb constructions (e.g., to, as in want to go).
- Verb – Action words or states of being (e.g., run, jump).
- WH-Word – Question words used to form interrogative and relative clauses (e.g., where, who).
- Existential there – The use of there to indicate the existence of something (e.g., there is a book on the table).

1.7.4. Syntactic operation

These features are derived from the penn-treebank syntactic parse of the stories. For example, the sentence “The cat sat on the mat” has the following Penn Treebank representation:

(S (NP (DT The) (NN cat)) (VP (VBD sat) (PP (IN on) (NP (DT the) (NN mat)))))

The number of closing nodes in a syntactic parse tree refers to the number of times a non-terminal node in the tree closes (i.e., when a constituent or subtree completes). This can be interpreted as the number of times a right parenthesis “)” appears in a Penn Treebank-style bracketed representation. Since each “)” represents the closure of a constituent, to extract the number of closing nodes from a Penn Treebank-style parse tree, we simply count the closing parentheses.

Similarly, the number of opening nodes in a syntactic parse tree refers to the number of times a new non-terminal node begins in a Penn Treebank-style representation. This corresponds to the number of times an opening parenthesis “(“ appears in the bracketed structure. Again, to compute the number of opening nodes, we simply count the number of closing parentheses.

Note that these features are intended to be general constructs in Linguistics, and not tied to a specific theory.

1.7.5. Syntactic state

These features are also derived from the Penn-Treebank syntactic parse of the stories.

We compute tree depth as the longest path from the root node to any leaf node. In a Penn Treebank-style parse tree, this corresponds to the maximum level of nested opening parentheses “(“ at any point in the string. The current depth at a given word is provided by subtracting the count of closed parentheses “)” from the number of open parentheses “(“.

For example, below is the depth count in square brackets for each word in the sentence.

```
(S
  (NP (DT The[2]) (NN cat[2]))
  (VP (VBD sat[2])
    (PP (IN on[3])
      (NP (DT the[4]) (NN mat[4]))
    )
  )
)
```

The number of open nodes at a particular word in a Penn Treebank-style parse tree corresponds to how many opening parentheses “(“ have been encountered without a matching closing parentheses “)” at that point in the string. In other words, it represents how many syntactic constituents are still "open" when the word appears in the tree.

**Step**	**Action**	**Open Nodes**	**Word Produced**
(S	1	**1**	-
(NP	1	**2**	-
(DT	1	**3**	-
The	(Word)	**3**	**The**
)	-1	**2**	-
(NN	1	**3**	-
cat	(Word)	**3**	**cat**
)	-1	**2**	-
)	-1	**1**	-
(VP	1	**2**	-
(VBD	1	**3**	-
sat	(Word)	**3**	**sat**
)	-1	**2**	-
(PP	1	**3**	-
(IN	1	**4**	-
on	(Word)	**4**	**on**
)	-1	**3**	-
(NP	1	**4**	-
(DT	1	**5**	-
the	(Word)	**5**	**the**
)	-1	**4**	-
(NN	1	**5**	-
mat	(Word)	**5**	**mat**
)	-1	**4**	-
)	-1	**3**	-
)	-1	**2**	-
)	-1	**1**	-
)	-1	**0**	- (End)

1.7.6. Semantic vector (word embedding)

We obtained 50-dimensional word embedding GloVe vectors (references 61,62) for each word in our dataset. These embeddings represent words as points in a high-dimensional space, capturing semantic and syntactic relationships based on co-occurrence patterns in large text corpora. The GloVe vectors are trained using a symmetric context window of size 10, and therefore they capture meaning beyond the single lexical item.

To reduce the dimensionality of these embeddings while preserving as much meaningful variation as possible, we applied Principal Component Analysis (PCA). PCA is a statistical technique that transforms the original high-dimensional data into a lower-dimensional space by identifying orthogonal directions (principal components) that capture the most variance in the data.

As a result of applying PCA, we extracted the top ten principal components. These components are ordered according to the amount of variance they explain in the original 50-dimensional GloVe embeddings. The first principal component captures the largest proportion of variance, followed by the second, and so on. This organization ensures that the most critical dimensions of variation in the word embeddings are retained, while less significant noise and redundancy are minimized.

By leveraging these ten principal components, we obtain a lower-dimensional representation of word meanings that retains the most important semantic distinctions while reducing computational complexity and mitigating redundancy in the original embedding space.

1.8. Back-to-back regression decoding

We fit a back-to-back regression algorithm [10], which allows us to decode multiple features from the MEG data while also controlling for their co-variation. The resulting model coefficients represent how robustly a linguistic feature is encoded in neural responses, above and beyond the variance accounted for by the other linguistic features.

For the neural decoding, the input features were the magnitude of activity at each of the 208 MEG sensors. This approach allows us to decode from multiple, potentially overlapping, neural representations, without relying on gross modulations in activation strength [10,11].

Because some of the features in our analysis are correlated with one another, we need to jointly evaluate the accuracy of each decoding model relative to its performance in predicting all modeled features, not just the target feature of interest. This is because, if fitting each feature independently, we will not be able to dissociate the decoding of feature *f* from the decoding of the correlated feature *f*ˆ. The necessity to use decoding over encoding models here, though (which, do not suffer so harshly from the problem of co-variance in the stimulus space) is one of signal to noise: we expect any signal related to linguistic processes to be contained in low-amplitude responses that are distributed over multiple sensors. Our chances of uncovering reliable responses to these features is boosted by using multivariate models.
To overcome the issue of covariance, but still to capitalize on the advantages of decoding approaches, we implement a back-to-back ridge regression model [30]. This involves a two stage process. First, a ridge regression model was fit on a random half of the data, at a single time-point (steps 1-6 in Figure 7). The mapping was learnt between the multivariate input (activity across sensors) and the univariate stimulus feature (one of the 54 features described above). All decoders were provided with data normalized by the mean and standard deviation in the training set:

$$arg\,min\beta \sum_i (y_i \beta^T X_i)^2 + \alpha||\beta||^2$$

where $y_i \in \{\pm1\}$ is the feature to be decoded at trial *i* and X_i is the multivariate neural measure.
The l2 regularization parameter α was also fit, testing 20 log-spaced values from 1^{-5} to 1^5. This was implemented using the *RidgeCV* function in *scikit-learn* [12].

Then, we use the other half of the acoustic or neural responses to generate a prediction for each of the 31 features corresponding to the test set. However, because the predictions are correlated, we need to jointly-evaluate the accuracy of decoding each feature, to take into account the variance explained by correlated non-target features. To do this, we fit another ridge regression model (steps 6-8 in Figure 7), this time learning the beta coefficients that map the matrix of *true* feature values to *predicted* feature values:

$$arg\,min\beta \sum_i (y_i \beta^T \hat{Y_i})^2 + \alpha||\beta||^2$$

where $y_i \in \{\pm1\}$ is the ground truth of a particular stimulus feature at trial i and $\hat{Y}_i$ is the prediction for all stimulus features. A new regularization parameter α was learnt for this stage. By including all stimulus features in the model, this accounts for the correlation between the feature of interest and the other features. From this, we use the beta-coefficients that maps the true stimulus feature to the predicted stimulus feature. Beta coefficients serve as our metric of decoding performance: if a stimulus features is not encoded in neural responses (the null hypothesis) then there will be no meaningful mapping between the true feature y and the model prediction $\hat{y}$. Thus, the beta coefficient will be zero – equivalent to chance performance. If, however, a feature *is* encoded in neural activity (the alternative hypothesis), we should uncover a significant relationship between y and $\hat{y}$, thus yielding an above-zero beta coefficient.

The train/test split was performed over 100 folds, and the beta-coefficients were averaged across folds. This circumvents the issue of unstable coefficients when modeling correlated variables. These steps were applied to each subject independently.

Each of these steps are depicted graphically below in Figure 7.

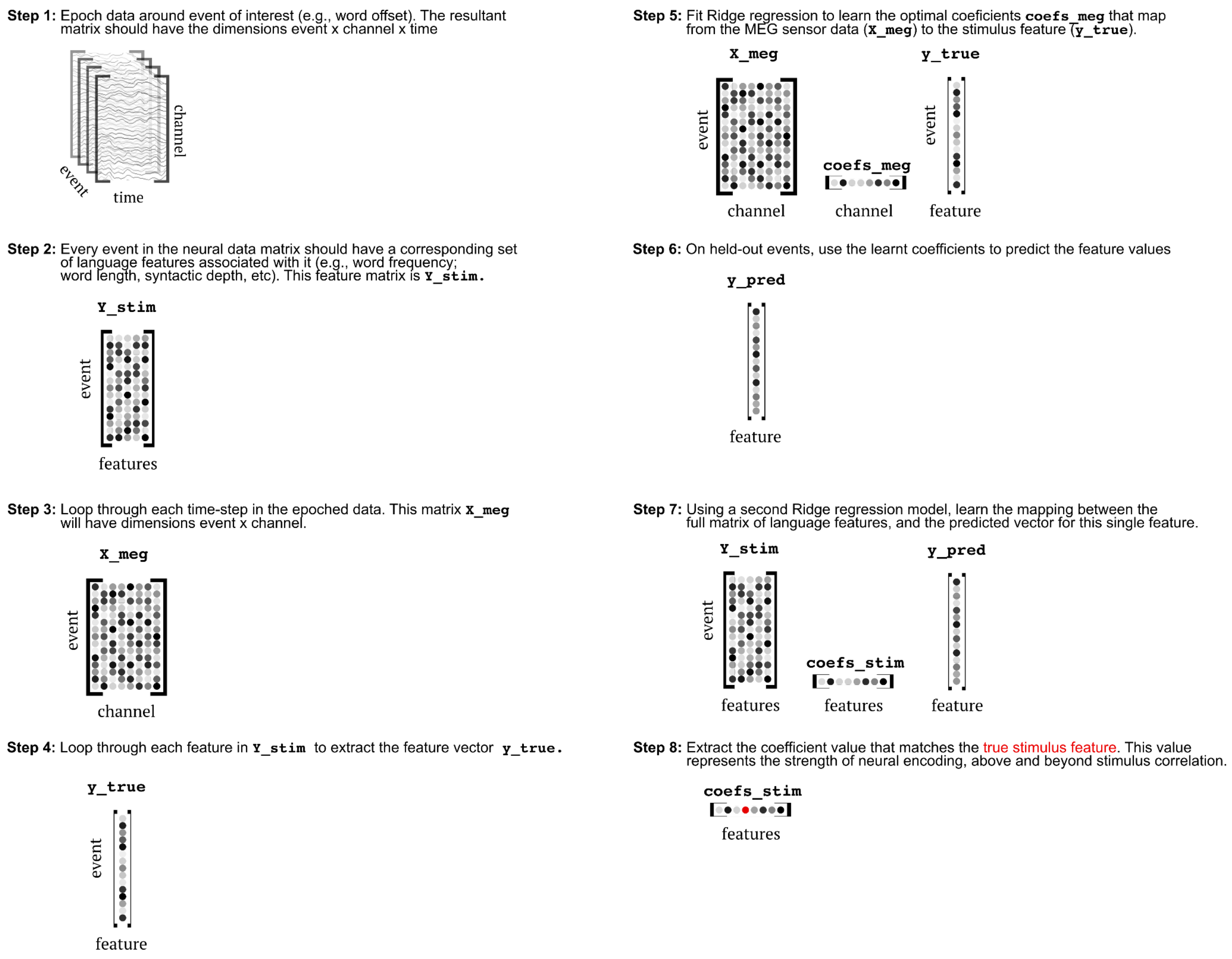

Figure 7: Steps of back-to-back regression analysis method.

1.9. Temporal generalization decoding

Temporal generalization (TG) consists of testing whether a temporal decoder fit on a training set at time t can decode a testing set at time t' [13]. This means that rather than evaluating decoding accuracy just at the

time sample that the model was trained on, we evaluate its accuracy across all possible train/testing time combinations.

TG can be summarized with a square training time × testing time decoding matrix. To quantify the stability of neural representations, we measured the duration of above-chance generalization of each temporal decoder. To quantify the dynamics of neural representations, we compared the mean duration of above-chance generalization across temporal decoders to the duration of above-chance temporal decoding (i.e. the diagonal of the matrix versus its rows). These two metrics were assessed within each subject and tested with second-level statistics across subjects.

1.10. Comparing decoding performance between trial subsets

To evaluate whether the processing of syntax built over time, we subset our analysis by evaluating decoding performance at different word positions in the sentence. We add a modification to our train/test cross-validation loop. The data are trained on the entire training set (i.e. the same number of trials as the 'typical analysis'), and the test set is grouped into the different levels of interest. We evaluate model performance separately on each split of the test data, which yields a time-course or generalization matrix for each group of trials that we evaluate on: in this case, each word position in the sentence.

1.11. Group statistics

To evaluate whether decoding performance is better than chance, we perform second- order statistics. This involves testing whether the distribution of beta coefficients across subjects significantly differs from chance (zero) across time using a one-sample permutation cluster test with default parameters specified in the MNE-Python package [7].

1.12. Simulation analyses

We simulated MEG responses to our story features by convolving the feature time series with temporally localized Gaussian kernels. Each feature (e.g., phonetic features, GloVe embeddings) is continuous, and the Gaussian was applied as a smoothing kernel across time, independently to each feature dimension. This simulates a scenario where neural responses follow an idealised, temporally smoothed version of the input feature.

Specifically, we created a Gaussian kernel with a peak time randomly sampled from a uniform distribution over a specified window (see Results), amplitude sampled uniformly from –1 to +1, and width sampled uniformly from a predefined range (see Results). These parameters allowed us to vary the temporal dynamics of the simulated response while preserving the structure and autocorrelation of the original features. No quantization or random sampling in feature space was performed — the original continuous feature vectors were preserved throughout. For specific values used in each simulation, see the corresponding parameters in the Results section. To approximate measurement variability, we added zero-mean Gaussian noise with a standard deviation of 0.2 to the final simulated MEG signals.

Because the feature time series for our variables (e.g., POS tags, semantic vectors) are defined at the word level, the input can be treated as a series of discrete impulses at word onset times. In practice, this means that the convolution with the Gaussian kernel is numerically equivalent to placing a scaled, temporally shifted Gaussian response at each word onset, then summing across words. This discrete implementation produces the same result as continuous convolution when the underlying feature is sparse in time. Thus, the resulting autocorrelation in the simulated MEG response reflects both the intrinsic structure of the input features and the overlap of these idealised neural responses.

To simulate a "static" response, we assigned the gaussian response encoding a given language feature to one MEG sensor. For a "dynamic" response, we encoded a given language feature in a sequence of gaussian responses, each spaced 50 ms apart.

To simulate the consequence of “destructive interference” under our different coding schemes, we simulated MEG responses as gaussian activation functions, with a peak response at 400 ms, amplitude of 1.5 femto-tesla, and response width equal to the average word duration in our stories (293 ms). We simulated responses of this static code using (i) exaggerated distance of 2 silent seconds between neighbouring words; (ii) actual distance between words from our story stimuli. To model maximal interference, we used a simulated feature vector that fluctuated between +1 and -1 at the onset of each word in the story. Finally, to simulate responses under the Hierarchical Dynamic Coding hypothesis, we encoded the maximally contrastive simulated feature in a sequence of gaussian responses that travel across space. Each gaussian in the sequence had a peak response at 400 ms, amplitude of 1.5 femto-tesla, and response width equal to the average word duration in our stories (293 ms). There were 3 gaussians in the sequence, occurring at 0 ms, 50 ms and 100 ms relative to feature onset.

Statistical analyses were performed by repeating the simulation procedure 1000 times and shuffling the correspondence between a given “neural generator” (the MEG sensor assigned to the feature) each time that feature was encountered.

All decoding analyses were performed in line with the analysis Methods described above, applied to the empirical MEG data.

REFERENCES

1. Ide, N. & Macleod, C. The american national corpus: A standardized resource of american english. in *Proceedings of corpus linguistics* vol. 3 1–7 (Lancaster University Centre for Computer Corpus Research on Language …, 2001).
2. Yuan, J. & Liberman, M. Speaker identification on the SCOTUS corpus. *J. Acoust. Soc. Am.* **123**, 3878–3878 (2008).
3. Gwilliams, L. *et al.* Introducing MEG-MASC a high-quality magneto-encephalography dataset for evaluating natural speech processing. *Sci Data* **10**, 862 (2023).
4. Fedorenko, E., Ivanova, A. A. & Regev, T. I. The language network as a natural kind within the broader landscape of the human brain. *Nat. Rev. Neurosci.* (2024) doi:10.1038/s41583-024-00802-4.
5. Ochshorn, R. M. & Hawkins, M. Gentle forced aligner. *github. com/lowerquality/gentle* (2017).
6. Adachi, Y., Shimogawara, M., Higuchi, M., Haruta, Y. & Ochiai, M. Reduction of non-periodic environmental magnetic noise in MEG measurement by continuously adjusted least squares method. *IEEE Trans. Appl. Supercond.* **11**, 669–672 (2001).
7. Gramfort, A. *et al.* MNE software for processing MEG and EEG data. *Neuroimage* **86**, 446–460 (2014).
8. King, S. & Taylor, P. Detection of phonological features in continuous speech using neural networks. *Comput. Speech Lang.* **14**, 333–353 (2000).
9. Balota, D. A. *et al.* The English Lexicon Project. *Behav. Res. Methods* **39**, 445–459 (2007).
10. King, J.-R., Charton, F., Lopez-Paz, D. & Oquab, M. Back-to-back regression: Disentangling the influence of correlated factors from multivariate observations. *Neuroimage* **220**, 117028 (2020).
11. King, J.-R., Gramfort, A. & Others. Encoding and decoding neuronal dynamics: Methodological framework to uncover the algorithms of cognition. (2018).
12. Pedregosa, F. *et al.* Scikit-learn: Machine Learning in Python. *arXiv [cs.LG]* 2825–2830 (2012).
13. King, J.-R. & Dehaene, S. Characterizing the dynamics of mental representations: the temporal generalization method. *Trends Cogn. Sci.* **18**, 203–210 (2014).

1. Supplementary Results

1.1. Distribution of word sequences across time

We observed that language features are maintained for a long time, potentially overlapping with the processing of many words into the future. To quantify the extent of this overlap, we computed the timing distribution of words in our stories.

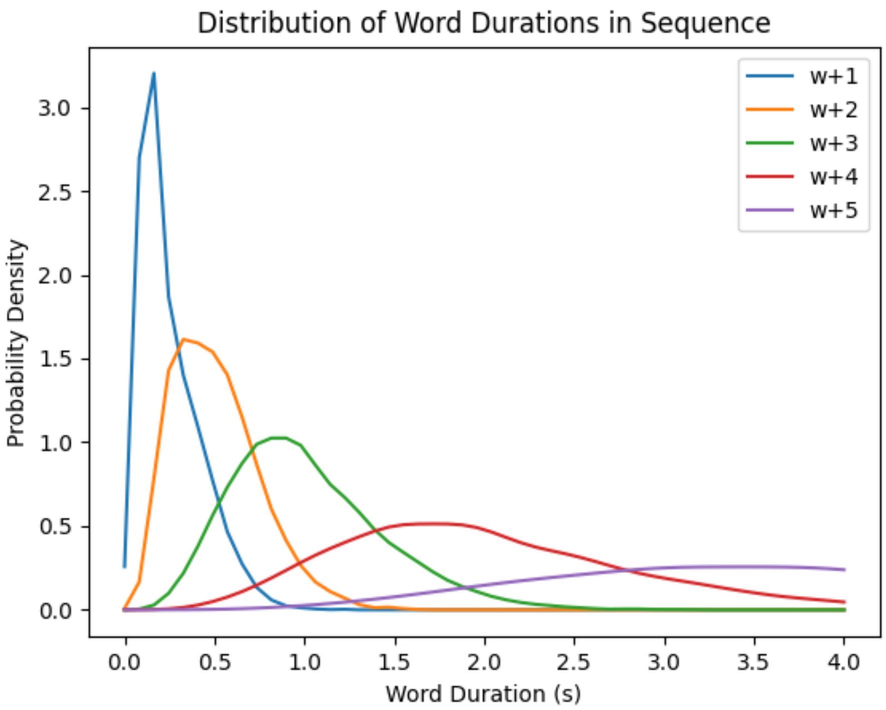

Figure 1: **Distribution of word duration.** Probability density function for the word onsets of the words 1, 2, 3, 4, and 5 words into the future sequence. This demonstrates that when a feature of w0 is maintained for 1 second into the future, this is, on average, being maintained into the processing of three or four words into the future.

1.2. *Hierarchy decoding at word onset*

We repeated the same analysis displayed in Main Figure 4A time-locked to word onset. The results of the permutation test are quantitatively weaker, but qualitatively comparable to the offset results. Phonetic features can be decoded from around 112:368 ms ($\hat{t}$ (average t-value in the cluster) = 1.96, p = .048); sub-lexical features from 80:304 ms ($\hat{t}$ = 1.56, p = .15); word class from 30:424 ms ($\hat{t}$ = 2.14, p = .013); syntactic operation from 232:944 ms ($\hat{t}$ = 2.33, p = .004); syntactic state ($\hat{t}$ = 3.57, p < .001) and semantic vectors ($\hat{t}$ = 3.97, p < .001) throughout the entire search window.

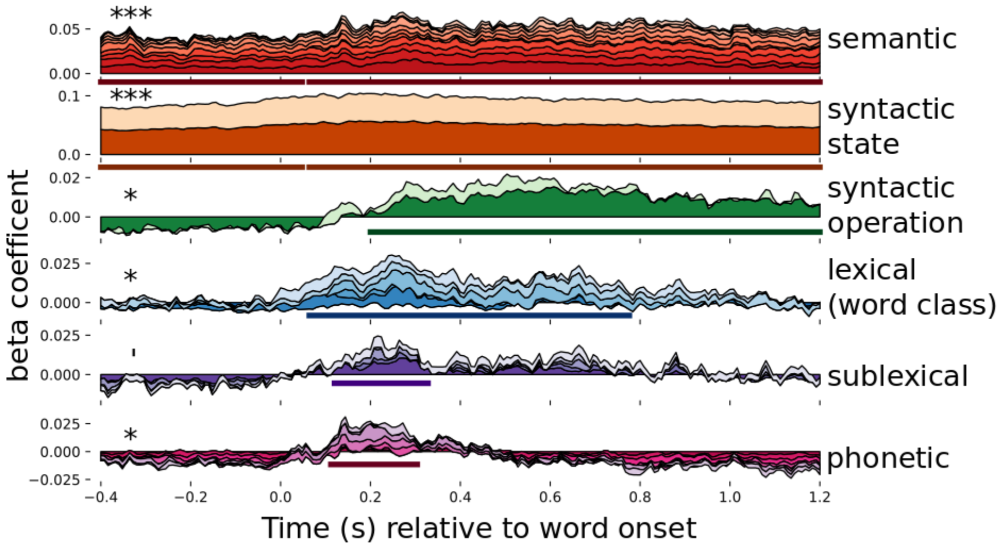

Figure 2: **Word onset language level decoding.** Result of decoding each language level over time when time locking the analysis to word onset. The beta coefficients of each feature are stacked on top of each other, such that the top of the timecourse plot corresponds to the cumulative sum of all features in that linguistic level. The x-axis corresponds to time in seconds relative to word offset. The y-axis corresponds to the cumulative beta-coefficient across features. Solid line below the time-course represents the extent of the significant temporal cluster; asterisks represent its significance: * p < .05; ** p < .01; *** p < .001.

1.3. Temporal Generalization Analysis Locked to Word-Onset

We repeated the same analysis displayed in Main Figure 5A time-locked to word onset. Decoding performance is numerically weaker, but the generalization pattern is similar to the word offset results: Wider diagonal pattern for increasing levels of the language hiearchy.

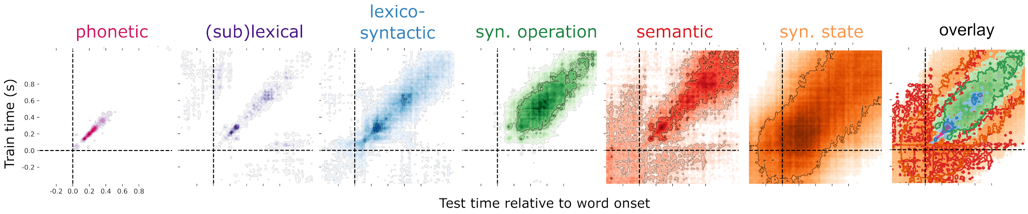

Figure 3: **Word onset temporal generalization.** Result of temporal generalization decoding at each language level over time when time locking the analysis to word onset.

1.4. Temporal permutation cluster extent at word onset and offset

While temporal cluster tests to not provide statistical assurance of the onset and offset times of an effect, we provide a summary of cluster times here for reference:

Table 1: Summary of cluster times locked to word onset and offset

**Level**	**Word Onset**	**Word Offset**
Phonetic	112:368 ms	-40:230 ms
Word Form	80:304 ms	-130:550 ms
Lexical-Syntactic	30:424 ms	-170:200 ms
Syntactic Operation	232:944 ms	-190:1200 ms
Syntactic State	-400:1200 ms	-400:1200 ms
Semantic	-400:1200 ms	-400:1200 ms

1.5. Replication across sessions

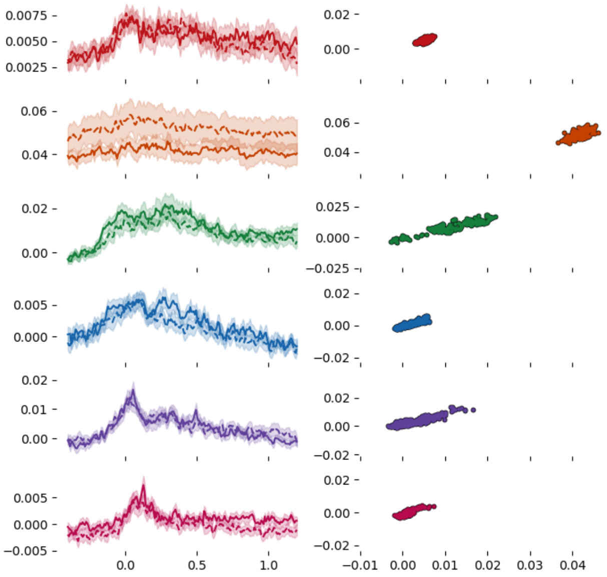

Figure 4: **Replicability across sessions.** Left: Average decoding accuracy is displayed for each of the six linguistic levels, from session one (dashed line) and session two (solid line). Right: Correlation between the average timecourses across subjects.

1.6. *Detailed decoding results for each linguistic feature*

If the decoding timecourses of a particular feature are significantly different from one another, we can use this as evidence for separable underlying processes. The results of this decoding time-locked to word offset is presented in Supplementary Figure 5, and relative to word onset is presented in Supplementary Figure 6 and displayed in each of the tables below.

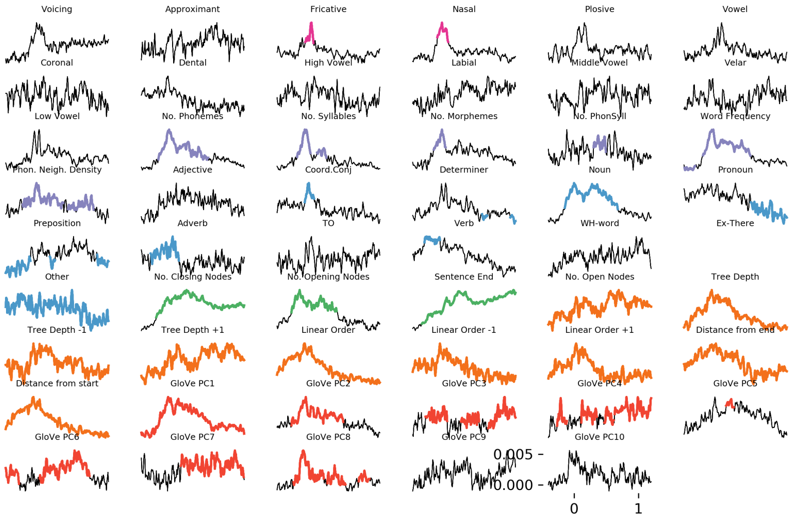

Figure 5: **Word offset feature decoding.** Average R-values across subjects for each of the linguistic features of interest, time-locked to word offset. Coloured portions of the time course correspond to temporal clusters that exceed a $p < .05$ threshold. Colour corresponds to the putative linguistic level.

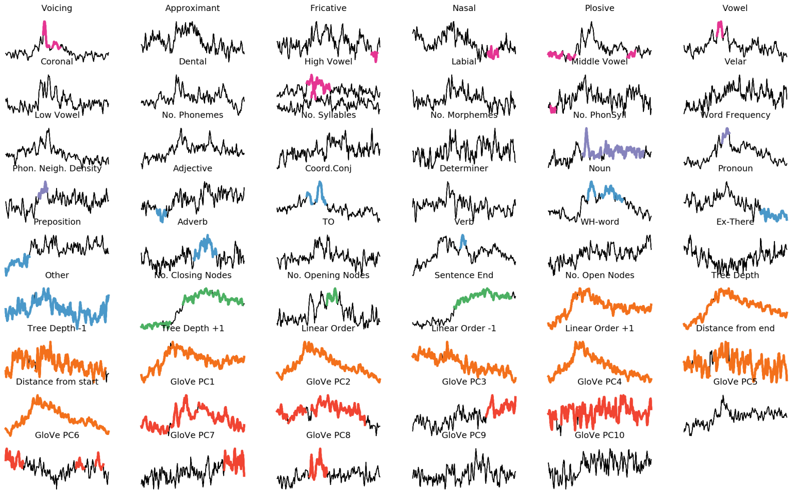

Figure 6: **Word onset feature decoding.** Average R-values across subjects for each of the linguistic features of interest, time-locked to word onset. Coloured portions of the time course correspond to temporal clusters that exceed a $p < .05$ threshold. Colour corresponds to the putative linguistic level.

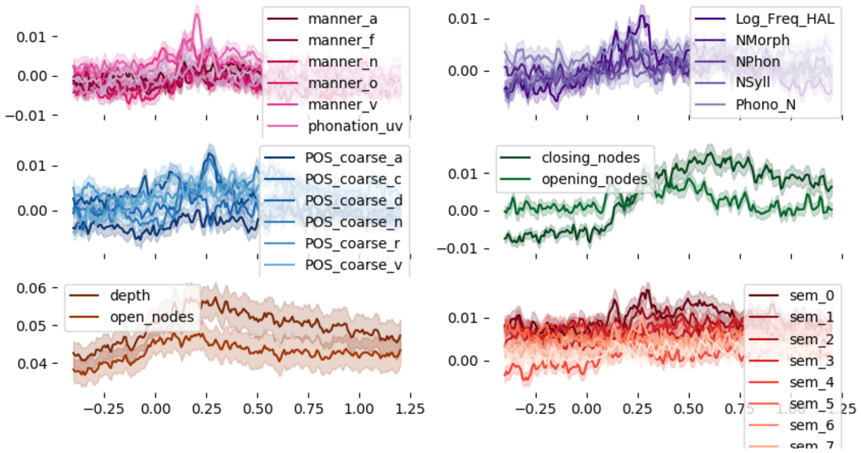

Figure 7: **Word onset feature decoding grouped.** Average R-values across subjects for each of the linguistic features of interest, time-locked to word onset, and grouped into feature families as shown in Figure 3. Coloured portions of the time course correspond to temporal clusters that exceed a $p < .05$ threshold. Colour corresponds to the putative linguistic level.

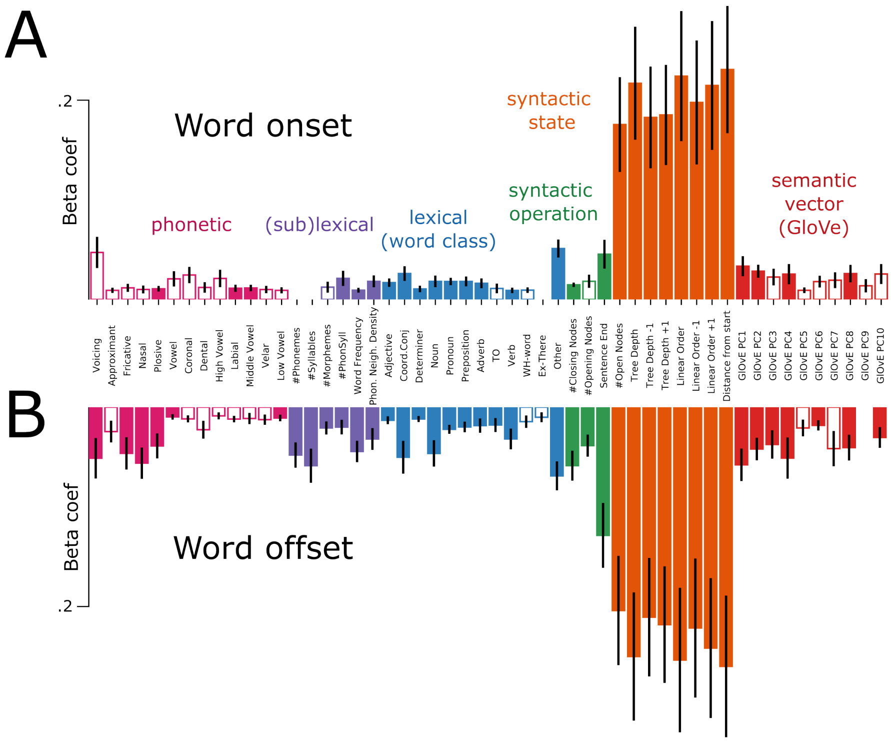

Figure 8: **Word onset and word offset average decoding performance.** Barplots represent average decoding over time, and error bars represent standard error of the mean. Filled bars correspond to features that are decodable significantly better than chance at p ¡ .05 level, confirmed with a random permutation test.

1.7. Simulations

In order to test the assumptions of the HDC hypothesis, we simulated the MEG data under different conditions. Below are the results of our simulations replicating main Figure 4.

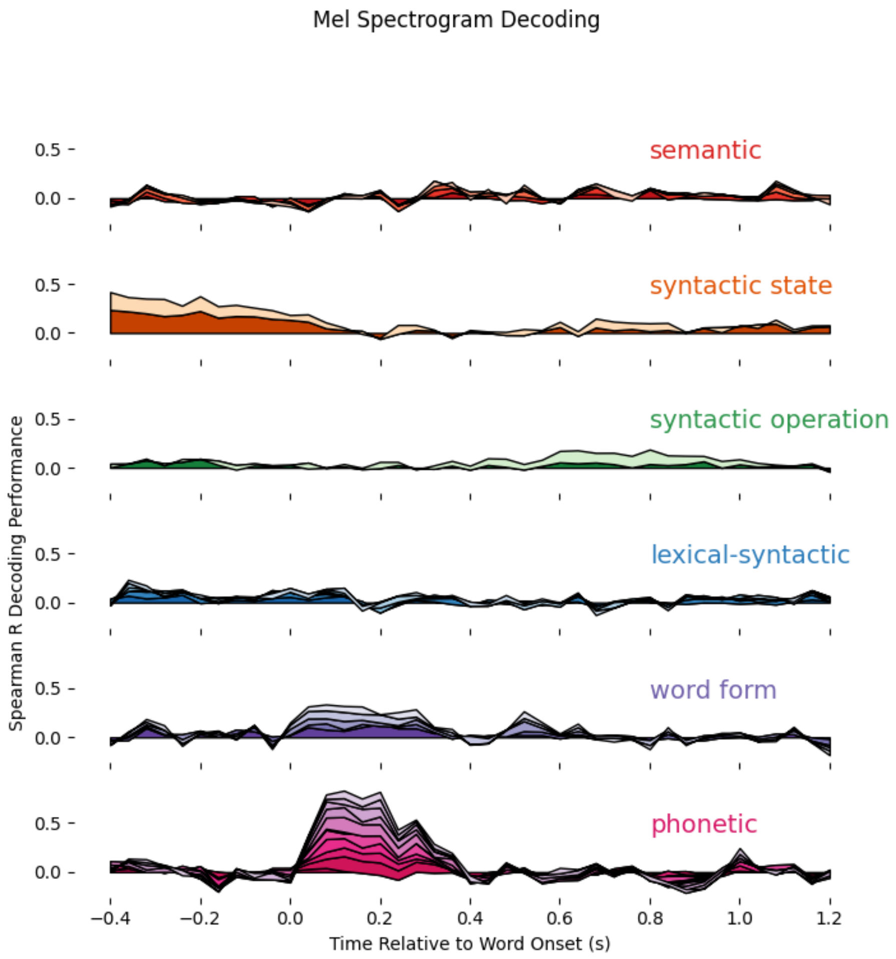

Figure 9: **Decoding language hierarchy from mel spectrogram.** The beta coefficients of each feature are stacked on top of each other, such that the top of the timecourse plot corresponds to the cumulative sum of all features in that linguistic level. The x-axis corresponds to time in seconds relative to word offset. The y-axis corresponds to the cumulative beta-coefficient across features.

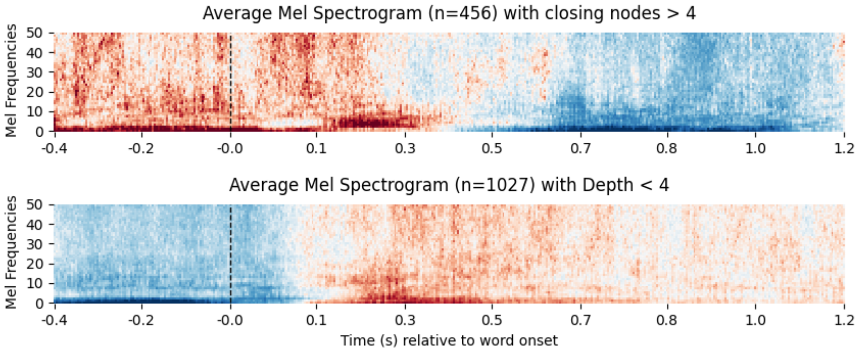

Figure 10: **Average Mel spectrogram for different language features.** We observed above-chance decoding for syntactic features from the Mel spectrogram, so we wanted to investigate the acoustic origin. We found that a large number of closing nodes are likely to occur at a sentence offset, thus leading to acoustic transitions into silence (above). Similarly, shallow sentence depth often occurs towards the beginning of a sentence, leading to systematic acoustic onsets (below).

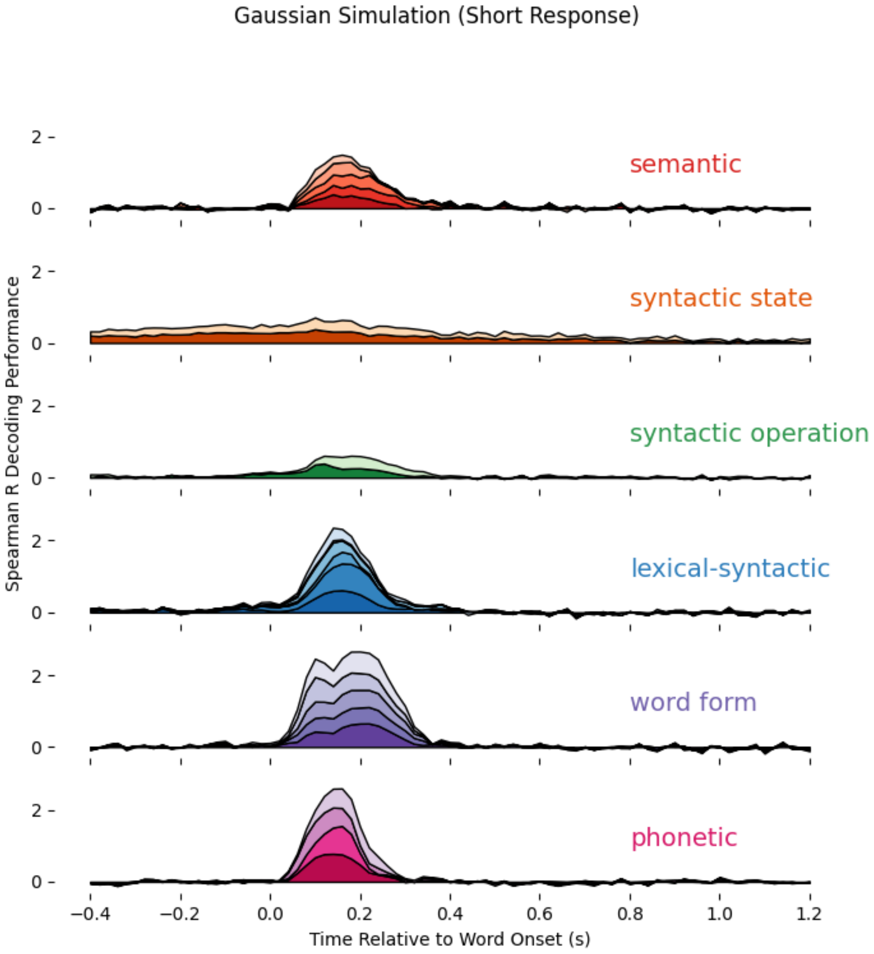

Figure 11: **Simulating MEG data as gaussian responses to language input.** The beta coefficients of each feature are stacked on top of each other, such that the top of the timecourse plot corresponds to the cumulative sum of all features in that linguistic level. The x-axis corresponds to time in seconds relative to word offset. The y-axis corresponds to the cumulative beta-coefficient across features.

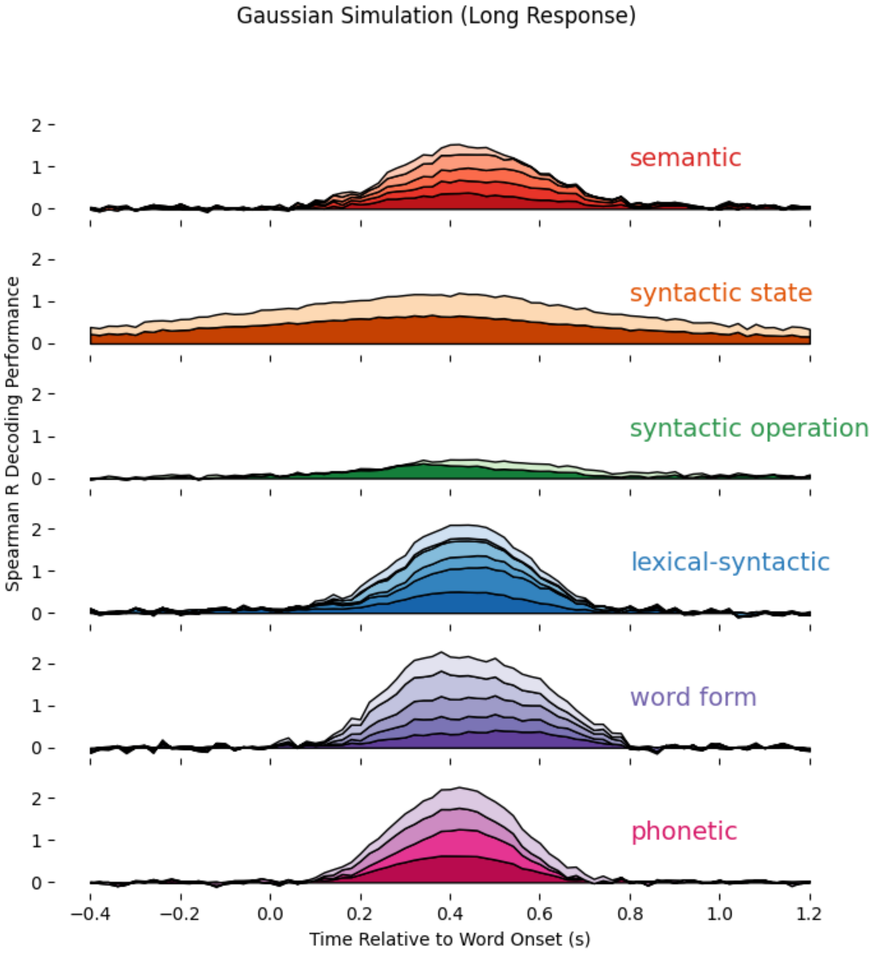

Figure 12: **Simulating MEG data as prolonged gaussian responses to language input.** The beta coefficients of each feature are stacked on top of each other, such that the top of the timecourse plot corresponds to the cumulative sum of all features in that linguistic level. The x-axis corresponds to time in seconds relative to word offset. The y-axis corresponds to the cumulative beta-coefficient across features.

1.8. Evoked feature responses

To connect our results to the comprehensive body of literature using ERP-style analyses, we also used regression encoding models to reconstruct the sensor-level responses to each of our stimulus features. Below we provide the full sensor response time-locked to word offset, as well as the root-mean-square of responses, when conducting median split of the trials.

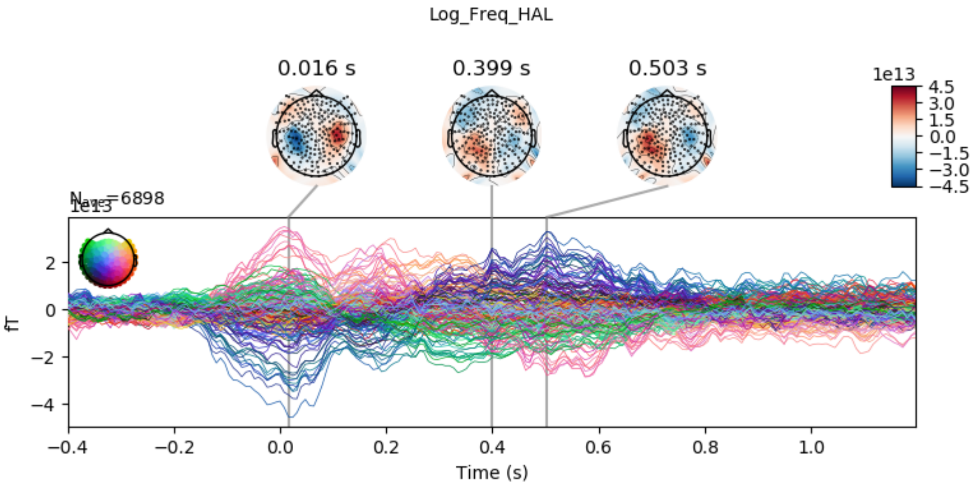

Figure 13: **Log Word Frequency sensor encoding.** x-axis represents time in seconds relative to word offset; y-axis represents the femto-tesla activity strength of each sensor.

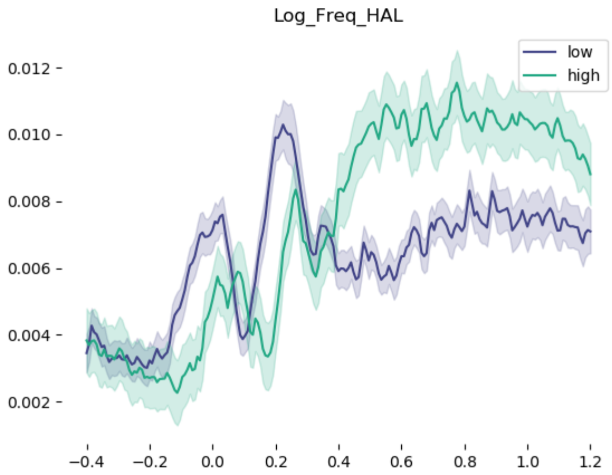

Figure 14: **Log Word Frequency RMS median split.** x-axis represents time in seconds relative to word offset; y-axis represents the root-mean-square of femto-tesla activity strength over all sensors.

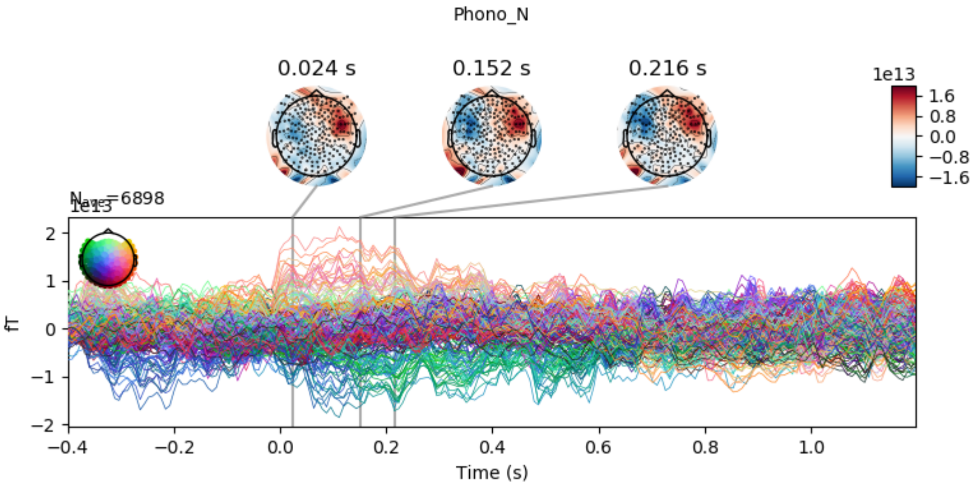

Figure 15: **Phonological Neighbourhood Density sensor encoding.** x-axis represents time in seconds relative to word offset; y-axis represents the femto-tesla activity strength of each sensor.

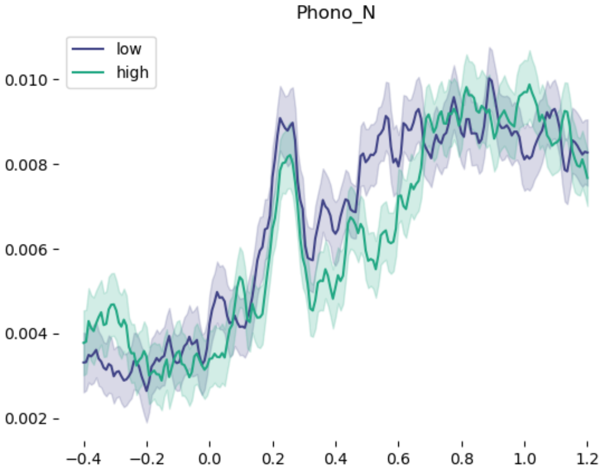

Figure 16: **Phonological Neighbourhood Density RMS median split.** x-axis represents time in seconds relative to word offset; y-axis represents the root-mean-square of femto-tesla activity strength over all sensors.

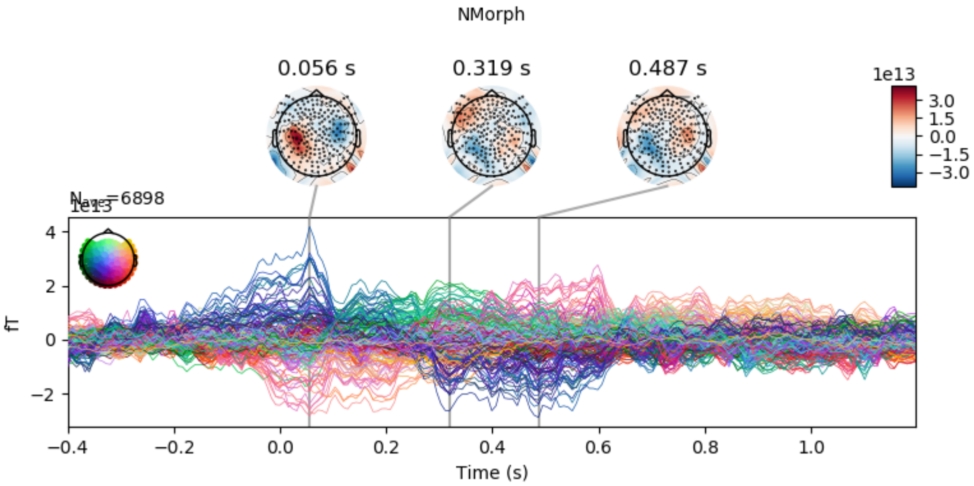

Figure 17: **Number of Morphemes sensor encoding.** x-axis represents time in seconds relative to word offset; y-axis represents the femto-tesla activity strength of each sensor.

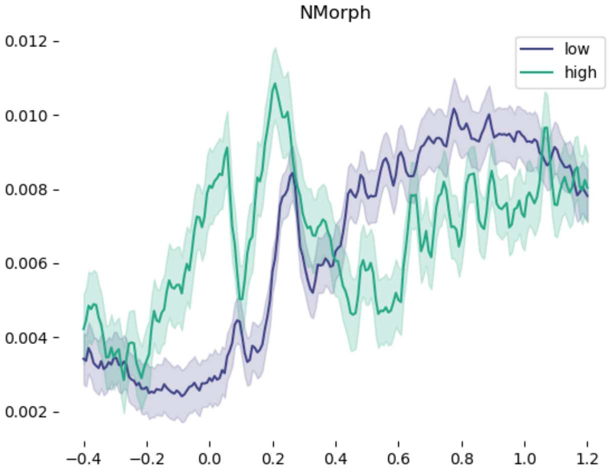

Figure 18: **Number of Morphemes RMS median split.** x-axis represents time in seconds relative to word offset; y-axis represents the root-mean-square of femto-tesla activity strength over all sensors.

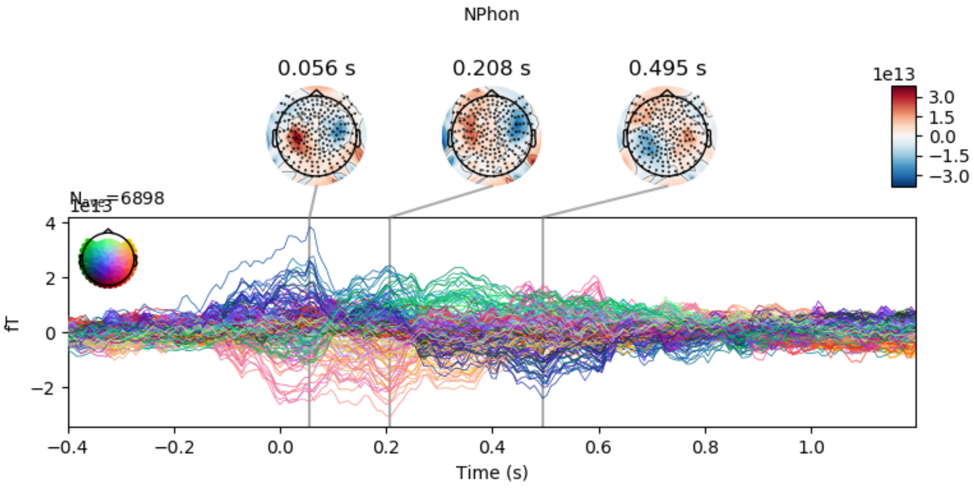

Figure 19: **Number of Phonemes sensor encoding.** x-axis represents time in seconds relative to word offset; y-axis represents the femto-tesla activity strength of each sensor.

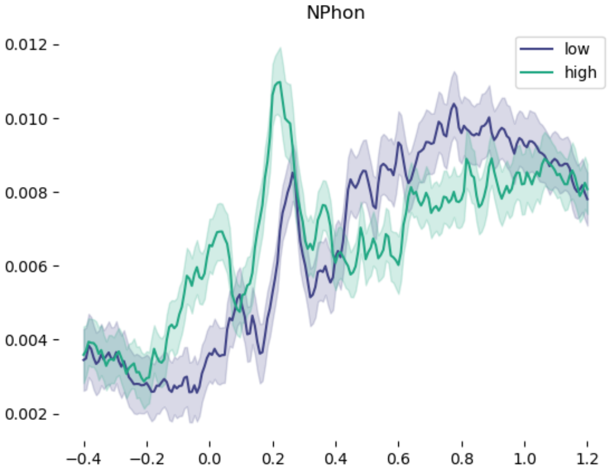

Figure 20: **Number of Phonemes RMS median split.** x-axis represents time in seconds relative to word offset; y-axis represents the root-mean-square of femto-tesla activity strength over all sensors.

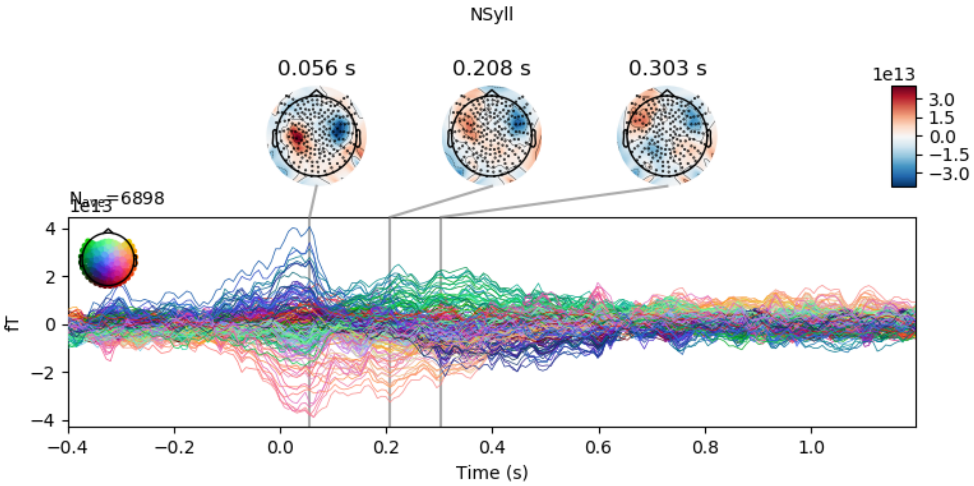

Figure 21: **Number of Syllables sensor encoding.** x-axis represents time in seconds relative to word offset; y-axis represents the femto-tesla activity strength of each sensor.

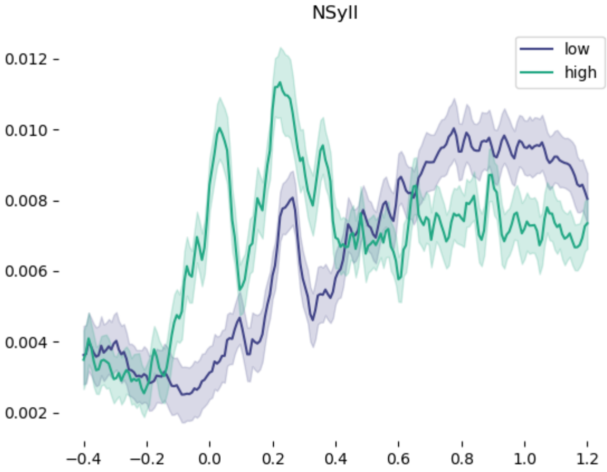

Figure 22: **Number of Syllables RMS median split.** x-axis represents time in seconds relative to word offset; y-axis represents the root-mean-square of femto-tesla activity strength over all sensors.

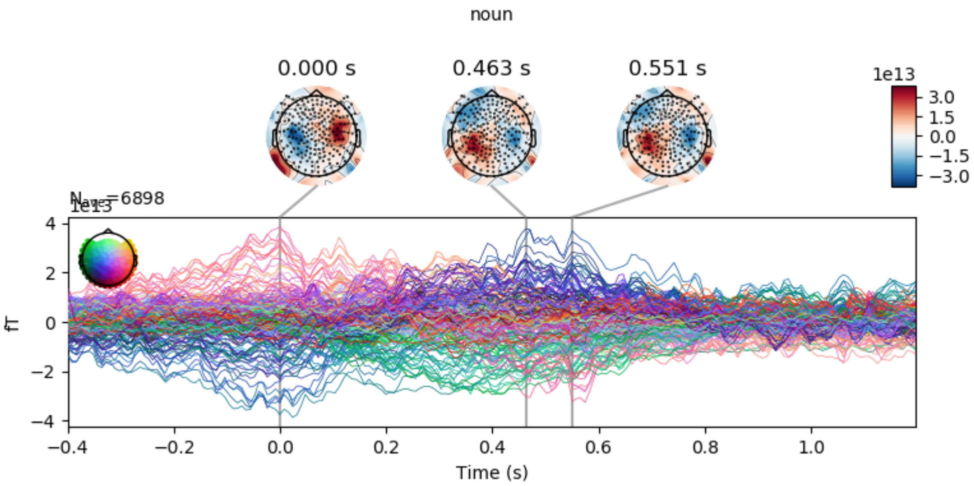

Figure 23: **Noun part-of-speech sensor encoding.** x-axis represents time in seconds relative to word offset; y-axis represents the femto-tesla activity strength of each sensor.

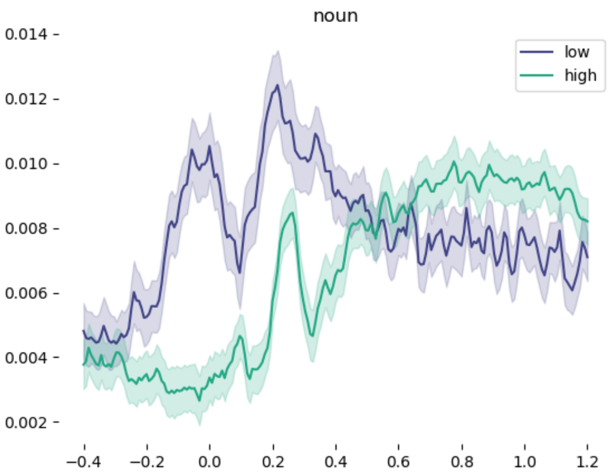

Figure 24: **Noun part-of-speech RMS median split.** x-axis represents time in seconds relative to word offset; y-axis represents the root-mean-square of femto-tesla activity strength over all sensors.

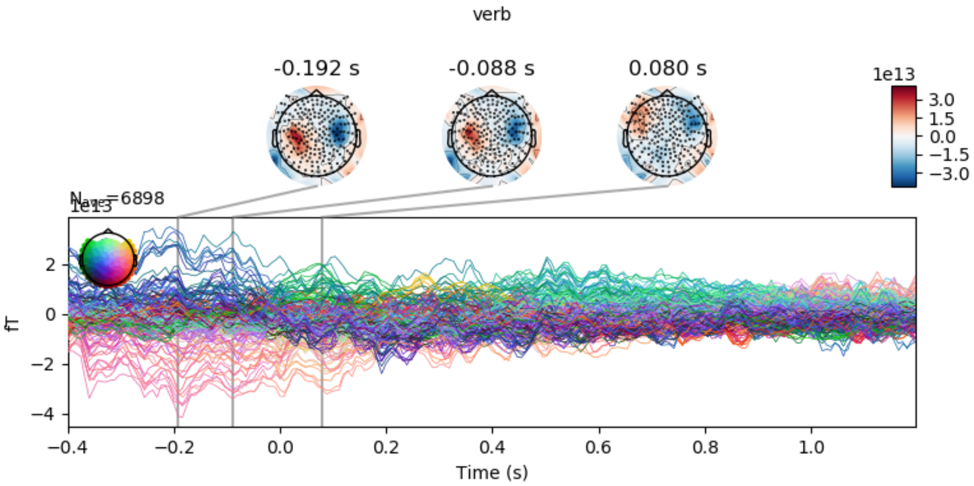

Figure 25: **Verb part-of-speech sensor encoding.** x-axis represents time in seconds relative to word offset; y-axis represents the femto-tesla activity strength of each sensor.

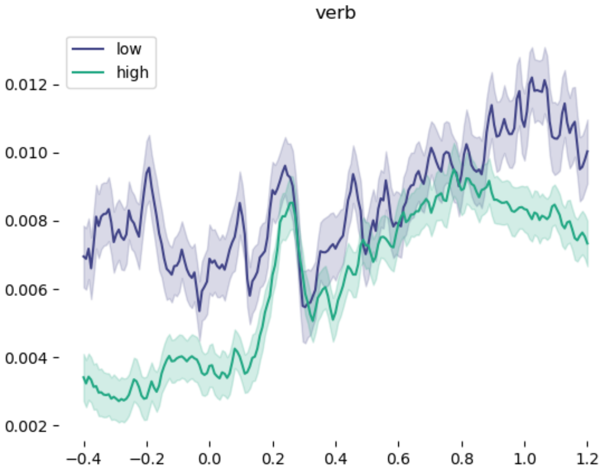

Figure 26: **Verb part-of-speech RMS median split.** x-axis represents time in seconds relative to word offset; y-axis represents the root-mean-square of femto-tesla activity strength over all sensors.

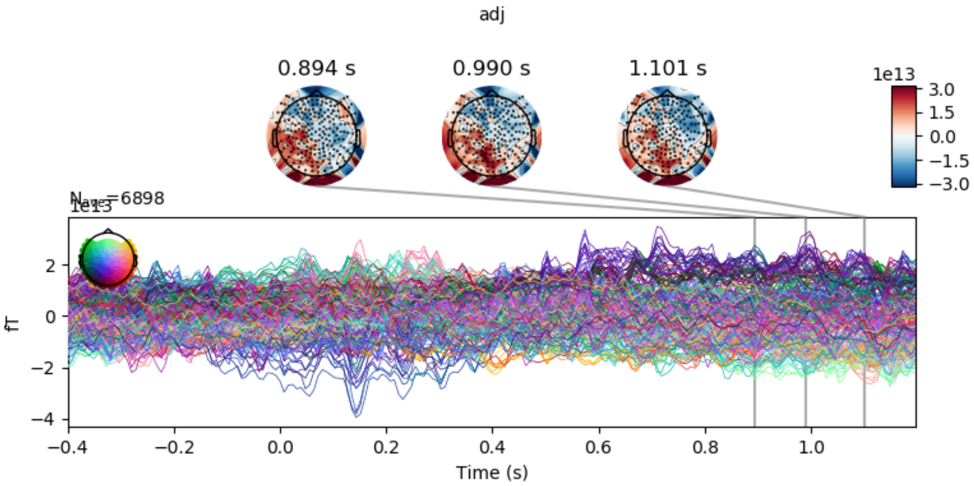

Figure 27: **Adjective part-of-speech sensor encoding.** x-axis represents time in seconds relative to word offset; y-axis represents the femto-tesla activity strength of each sensor.

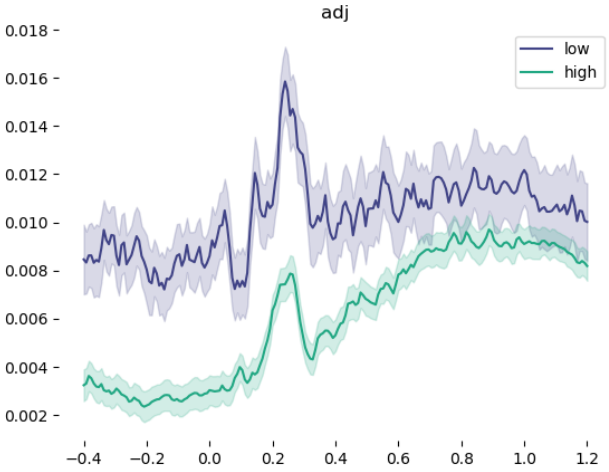

Figure 28: **Adjective part-of-speech RMS median split.** x-axis represents time in seconds relative to word offset; y-axis represents the root-mean-square of femto-tesla activity strength over all sensors.

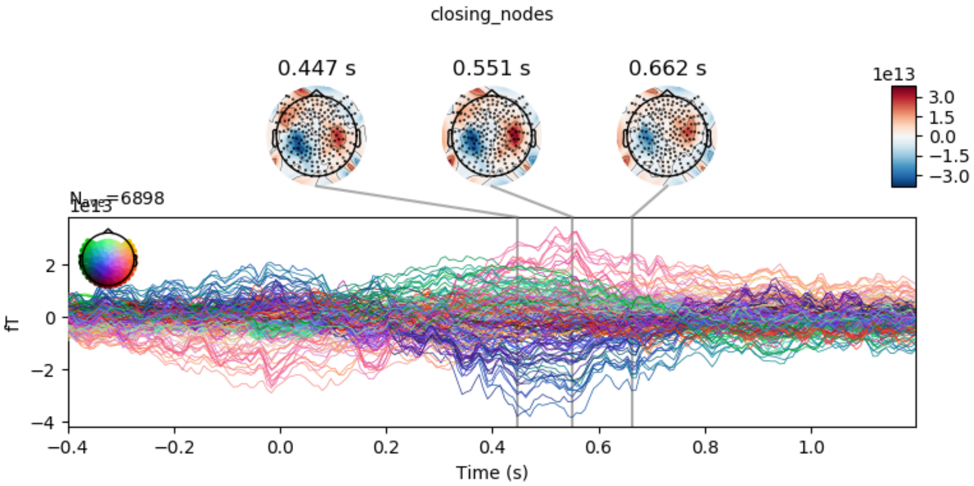

Figure 29: **Number of closing nodes sensor encoding.** x-axis represents time in seconds relative to word offset; y-axis represents the femto-tesla activity strength of each sensor.

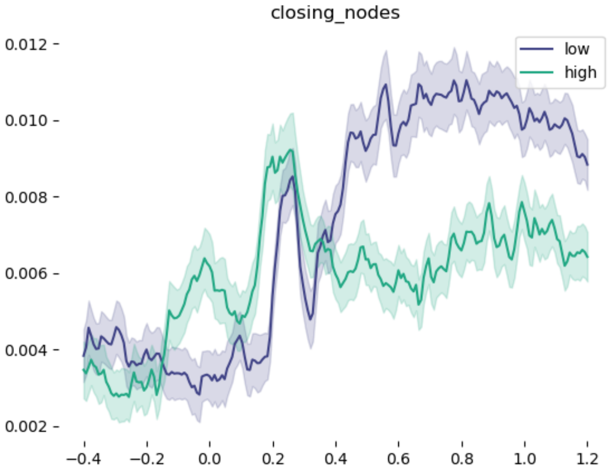

Figure 30: **Number of closing nodes RMS median split.** x-axis represents time in seconds relative to word offset; y-axis represents the root-mean-square of femto-tesla activity strength over all sensors.

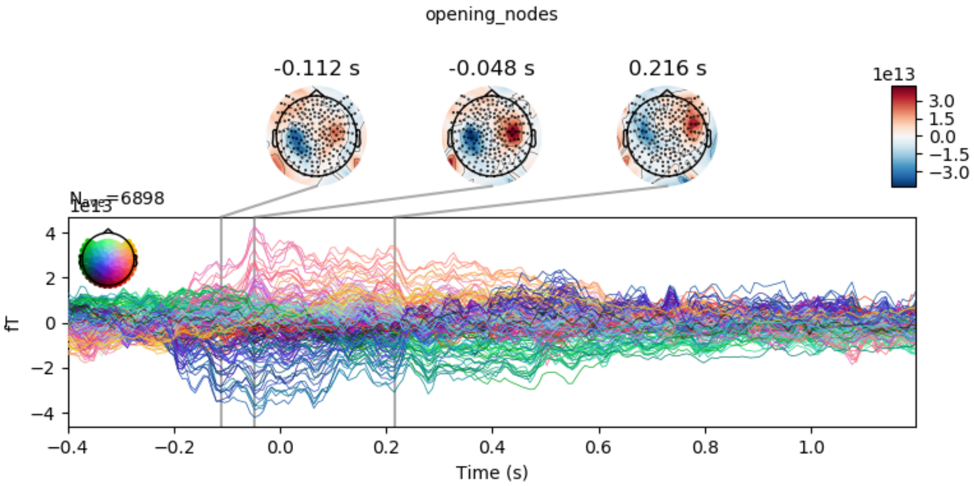

Figure 31: **Number of opening nodes sensor encoding.** x-axis represents time in seconds relative to word offset; y-axis represents the femto-tesla activity strength of each sensor.

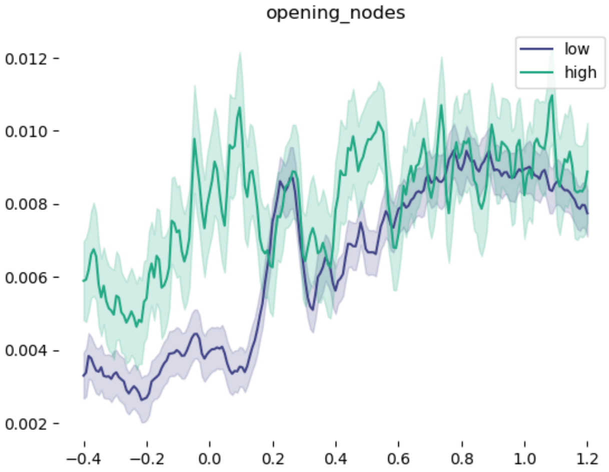

Figure 32: **Number of opening nodes RMS median split.** x-axis represents time in seconds relative to word offset; y-axis represents the root-mean-square of femto-tesla activity strength over all sensors.

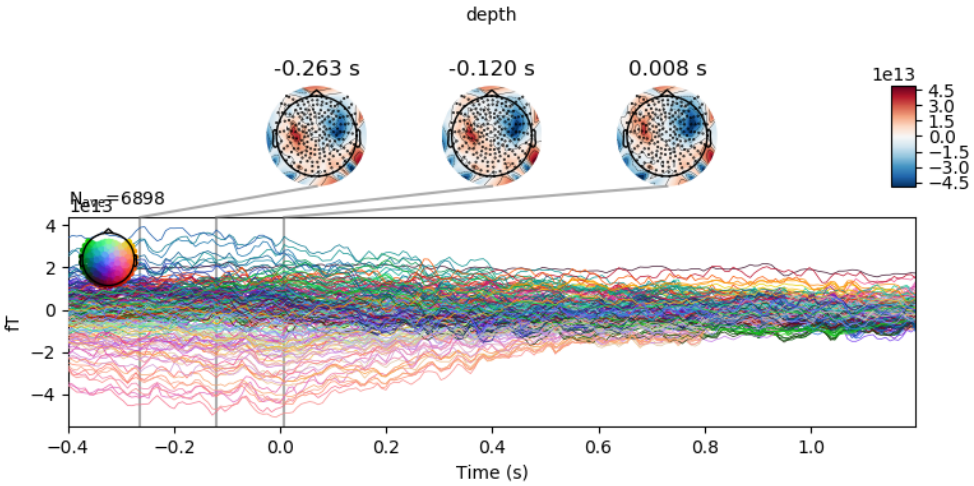

Figure 33: **Tree depth sensor encoding.** x-axis represents time in seconds relative to word offset; y-axis represents the femto-tesla activity strength of each sensor.

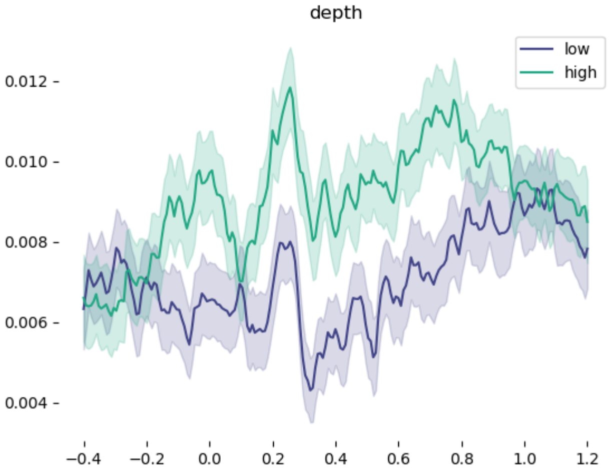

Figure 34: **Tree depth RMS median split.** x-axis represents time in seconds relative to word offset; y-axis represents the root-mean-square of femto-tesla activity strength over all sensors.

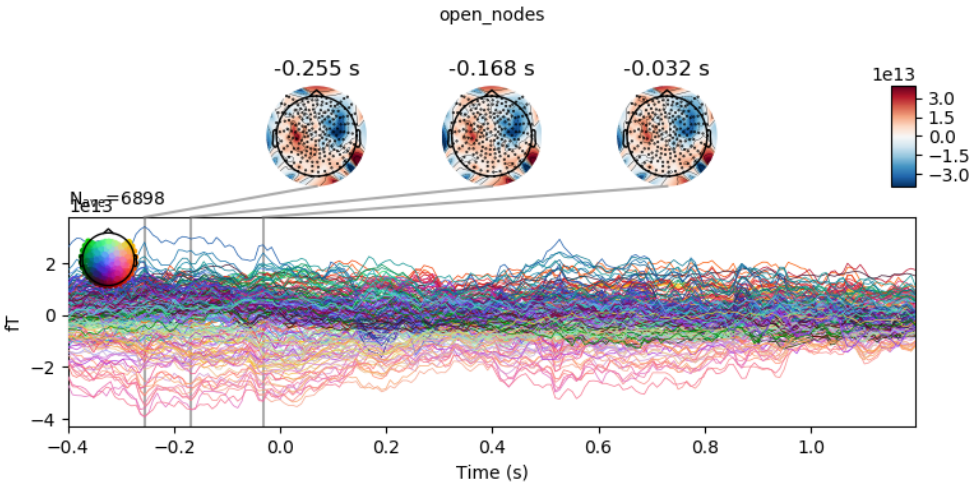

Figure 35: **Open Nodes sensor encoding.** x-axis represents time in seconds relative to word offset; y-axis represents the femto-tesla activity strength of each sensor.

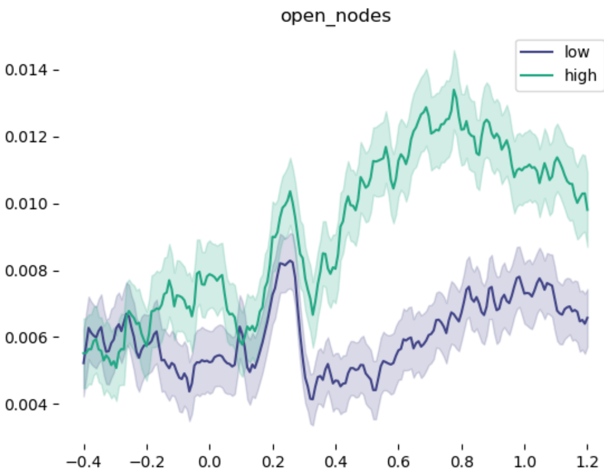

Figure 36: **Open Nodes RMS median split.** x-axis represents time in seconds relative to word offset; y-axis represents the root-mean-square of femto-tesla activity strength over all sensors.

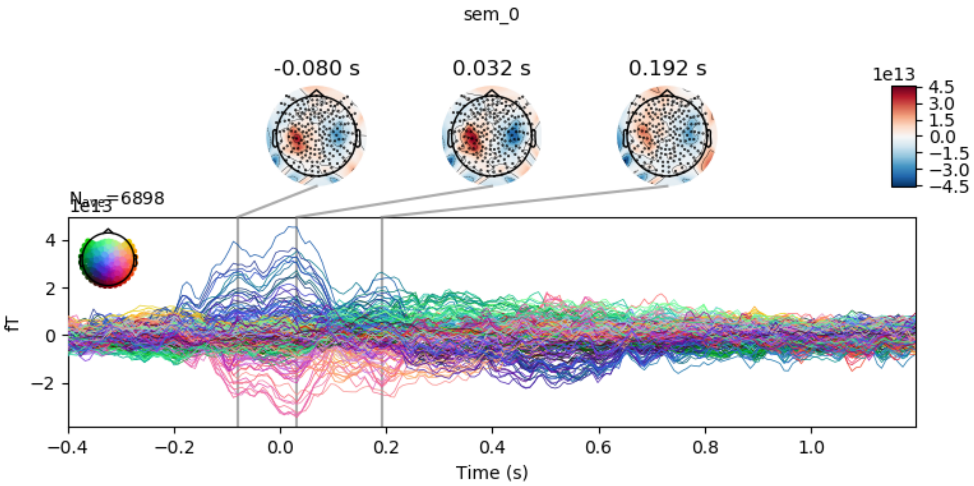

Figure 37: **GloVe PC1 sensor encoding.** x-axis represents time in seconds relative to word offset; y-axis represents the femto-tesla activity strength of each sensor.

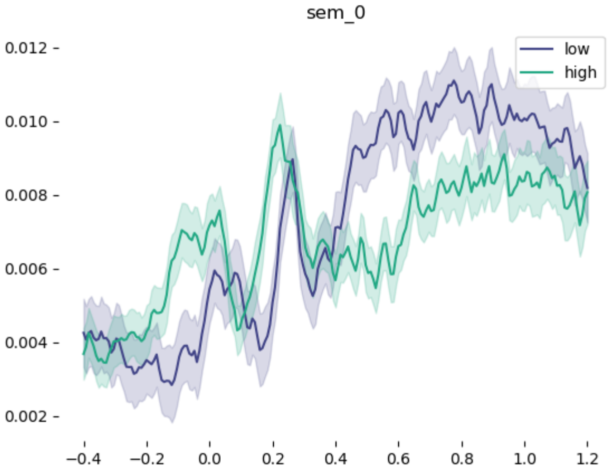

Figure 38: **GloVe PC1 RMS median split.** x-axis represents time in seconds relative to word offset; y-axis represents the root-mean-square of femto-tesla activity strength over all sensors.

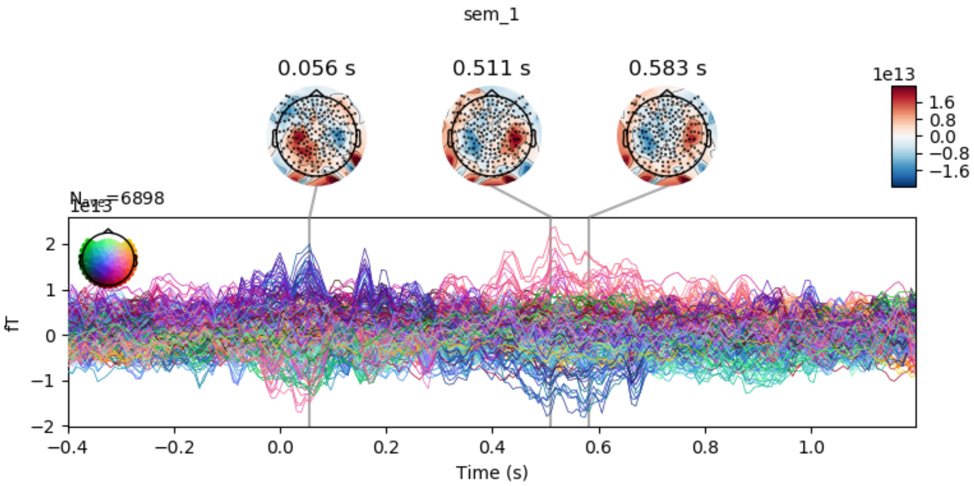

Figure 39: **GloVe PC2 sensor encoding.** x-axis represents time in seconds relative to word offset; y-axis represents the femto-tesla activity strength of each sensor.

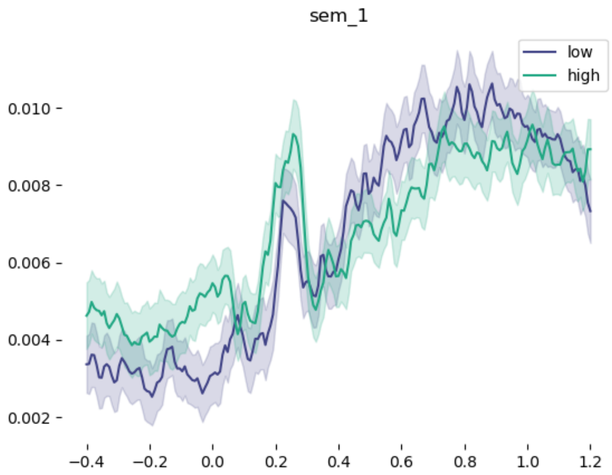

Figure 40: **GloVe PC2 RMS median split.** x-axis represents time in seconds relative to word offset; y-axis represents the root-mean-square of femto-tesla activity strength over all sensors.

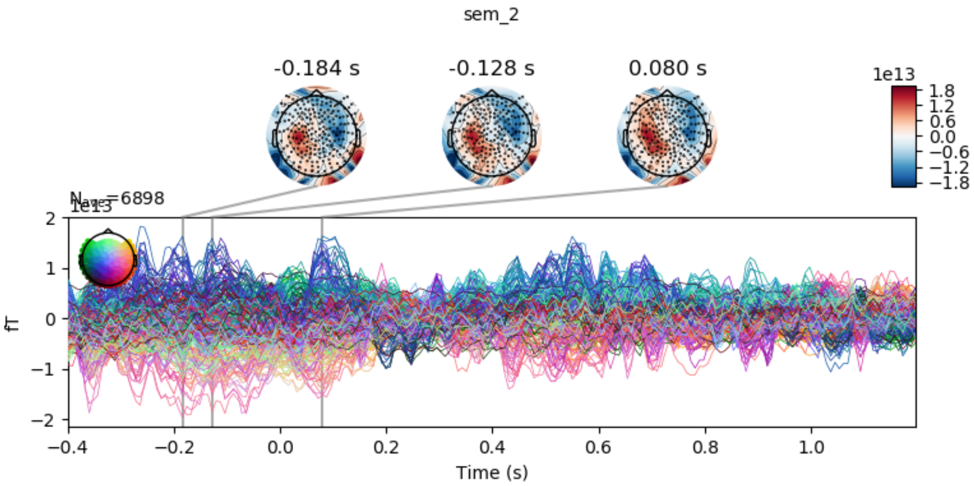

Figure 41: **GloVe PC3 sensor encoding.** x-axis represents time in seconds relative to word offset; y-axis represents the femto-tesla activity strength of each sensor.

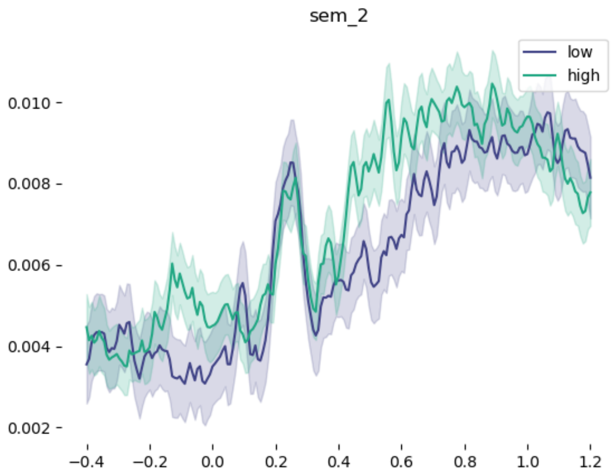

Figure 42: **GloVe PC3 RMS median split.** x-axis represents time in seconds relative to word offset; y-axis represents the root-mean-square of femto-tesla activity strength over all sensors.

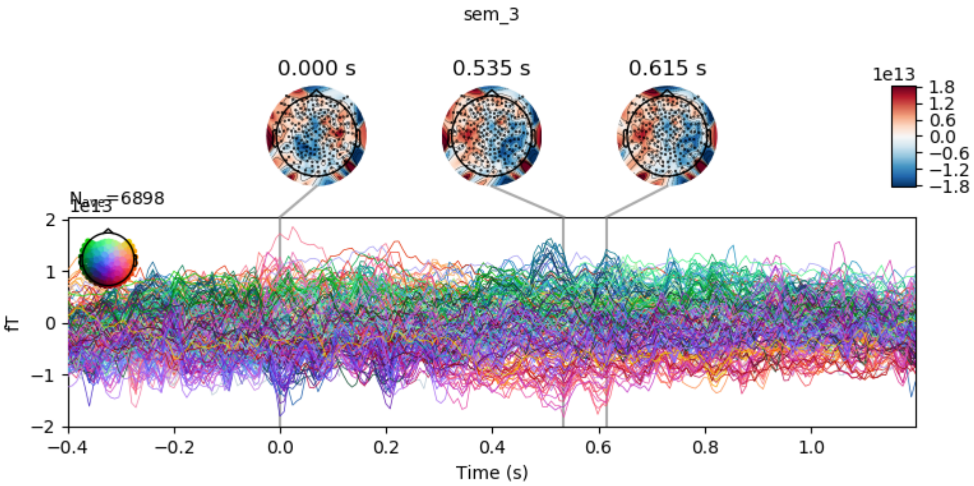

Figure 43: **GloVe PC4 sensor encoding.** x-axis represents time in seconds relative to word offset; y-axis represents the femto-tesla activity strength of each sensor.

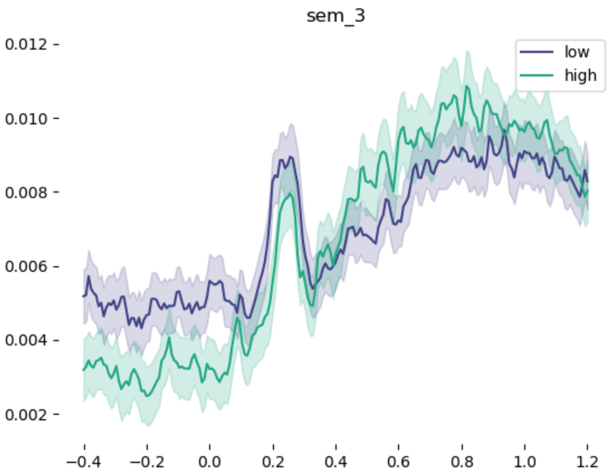

Figure 44: **GloVe PC4 RMS median split.** x-axis represents time in seconds relative to word offset; y-axis represents the root-mean-square of femto-tesla activity strength over all sensors.

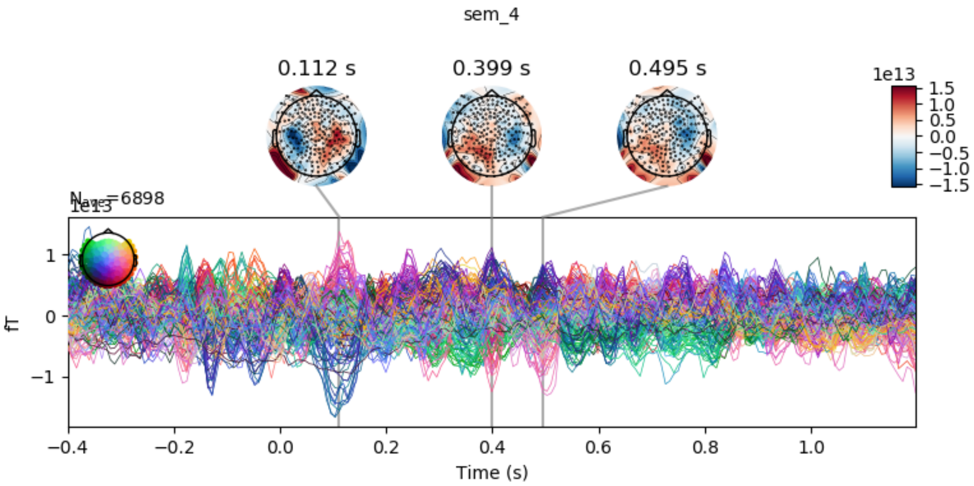

Figure 45: **GloVe PC5 sensor encoding.** x-axis represents time in seconds relative to word offset; y-axis represents the femto-tesla activity strength of each sensor.

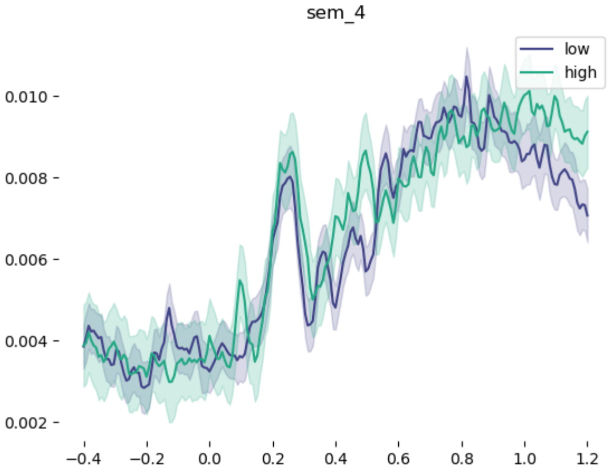

Figure 46: **GloVe PC5 RMS median split.** x-axis represents time in seconds relative to word offset; y-axis represents the root-mean-square of femto-tesla activity strength over all sensors.

Feature	$\hat{t}$	p	Window
Approximant	all p > .1		
Fricative	2.78	0.0245	39:168
Nasal	3.91	0.0065	-16:152
Plosive	all p > .1		
Vowel	-2.65	0.0917	1032:1088
Voicing	-2.34	0.0981	-392:-328
Coronal	None		
Dental	all p > .1		
High Vowel	all p > .1		
Labial	all p > .1		
Middle Vowel	None		
Velar	all p > .1		
Low Vowel	all p > .1		

Supplementary Table 1. Results of temporal permutation cluster test applied to the phonetic features relative to word offset.

Feature	$\hat{t}$	p	Window
Word Frequency	4.25	0.0001	-104:632
Word Frequency	-3.18	0.0136	-392:-224
No. Morphemes	3.56	0.0045	-72:111
No. Morphemes	3.1	0.0649	303:360
No. Phonemes	4.18	0.0001	-136:640
No. PhonSyll	2.63	0.0815	-128:-64
No. PhonSyll	2.67	0.016	303:496
No. Syllables	4.03	0.0061	-88:120
No. Syllables	2.9	0.0271	223:368
Phon. Neigh. Density	3.2	0.0008	-136:496
Phon. Neigh. Density	2.37	0.0432	568:680
Phon. Neigh. Density	2.79	0.0079	704:968

Supplementary Table 2. Results of temporal permutation cluster test applied to the sub-lexical features relative to word offset.

Feature	$\hat{t}$	p	Window
Adjective	all p > .1		
Coord.Conj	3.34	0.0125	31:176
Determiner	-2.67	0.062	-288:-224
Determiner	-2.49	0.0465	680:759
Determiner	-2.9	0.0354	1120:1200
Noun	4.88	0.0001	-144:680
Pronoun	-2.71	0.0509	544:616
Pronoun	-4.08	0.0001	640:1200
Preposition	-4.15	0.0001	-400:-16
Preposition	-2.59	0.0875	31:87
Preposition	-3.54	0.025	288:368
Preposition	-3.64	0.0039	1024:1200
Adverb	2.44	0.0638	-400:-320
Adverb	3.17	0.0015	-256:183
TO	all p > .1		
Verb	2.91	0.0055	-216:23
Verb	-2.85	0.0812	1000:1056
WH-word	None		
Ex-There	None		

Supplementary Table 3. Results of temporal permutation cluster test applied to the word class features relative to word offset.

Feature	$\hat{t}$	p	Window
No. Closing Nodes	5.82	0.0001	-168:1200
No. Opening Nodes	3.47	0.0001	-176:528
Sentence End	6.22	0.0001	-256:1200

Supplementary Table 4. Results of temporal permutation cluster test applied to the syntactic operation features relative to word offset.

Feature	$\hat{t}$	p	Window
Tree Depth	11.16	0.0001	-400:1200
Tree Depth -1	9.63	0.0001	-400:1200
Tree Depth +1	10.91	0.0001	-400:1200
No. Open Nodes	10.52	0.0001	-400:1200
Linear Order	11.74	0.0001	-400:1200
Distance from end	10.19	0.0001	-400:1200
Linear Order -1	9.64	0.0001	-400:1200
Linear Order +1	9.85	0.0001	-400:1200
Distance from start	10.93	0.0001	-400:1200

Supplementary Table 5. Results of temporal permutation cluster test applied to the syntactic state features relative to word offset.

Feature	$\hat{t}$	p	Window
GloVe PC1	5.91	0.0001	-400:1200
GloVe PC2	3.45	0.001	-176:632
GloVe PC3	2.82	0.0625	-320:-248
GloVe PC3	2.86	0.0095	-200:136
GloVe PC3	2.63	0.0145	352:632
GloVe PC3	2.31	0.085	672:736
GloVe PC3	2.79	0.0064	775:1200
GloVe PC4	2.54	0.0895	-368:-304
GloVe PC4	2.96	0.0343	-264:-111
GloVe PC4	3.02	0.0194	111:352
GloVe PC4	2.6	0.0666	368:455
GloVe PC4	2.62	0.0412	472:616
GloVe PC4	3.23	0.0054	632:1200
GloVe PC5	2.7	0.0859	55:111
GloVe PC5	2.7	0.0281	256:376
GloVe PC5	2.57	0.0661	463:536
GloVe PC6	2.89	0.0142	-400:-192
GloVe PC6	2.63	0.0931	0:55
GloVe PC6	3.27	0.0008	128:904
GloVe PC7	2.97	0.0881	-400:-344
GloVe PC7	3.18	0.0004	215:1192
GloVe PC8	3.98	0.0018	-144:327
GloVe PC8	2.69	0.0107	384:664
GloVe PC8	2.66	0.0269	872:1040
GloVe PC9	all p > .1		
GloVe PC10	all p > .1		

Supplementary Table 6. Results of temporal permutation cluster test applied to the semantic word embedding features relative to word offset.

1.9. Replication of reverse hierarchy across sessions

In order to assess the reliability of our results, we re-ran the hierarchical analysis on just the first and on just the second session of data separately (Figure 4). We find that the results are very similar across sessions, yielding a significant correlation between the averaging decoding timecourse of sessions 1 and 2 (Phonetic: $r = 0.68$, $p < .001$; Sub-lexical: $r = 0.86$, $p < .001$; Lexical: $r = 0.89$, $p < .001$; Syntactic operation: $r = 0.86$, $p < .001$; Syntactic state: $r = 0.62$, $p < .001$; Semantic: $r = 0.68$, $p < .001$).

Feature	$\hat{t}$	p	Window
Approximant		all p > .1	
Fricative	-2.52	0.0473	1072:1160
Nasal	-3.09	0.0522	600:672
Nasal	-2.49	0.095	688:752
Nasal	-2.77	0.0118	767:928
Plosive	-2.54	0.0358	-400:-304
Plosive	-2.85	0.0236	-288:-184
Plosive	-3.13	0.0649	-168:-111
Plosive	-3.29	0.015	-96:16
Plosive	-2.54	0.0225	832:952
Vowel	3.08	0.0363	111:199
Vowel	2.88	0.0658	215:280
Voicing	3.33	0.0378	183:280
Voicing	2.7	0.0379	327:447
Coronal	3.36	0.0881	248:296
Dental	3.23	0.0873	136:183
Glottal	2.62	0.0635	-88:0
Glottal	2.83	0.0106	64:424
Glottal	2.34	0.0932	504:568
Glottal	2.58	0.0954	584:640
Glottal	2.61	0.0776	712:783
Glottal	2.31	0.0952	936:1000
High Vowel	2.97	0.0492	136:199
Labial		all p > .1	
Middle Vowel	-2.51	0.0398	-368:-288
Velar		all p > .1	
Low Vowel	3.06	0.0595	231:288

Supplementary Table 7. Results of temporal permutation cluster test applied to the phonetic features relative to word onset.

Feature	$\hat{t}$	p	Window
Word Frequency	3.44	0.0107	191:303
Word Frequency	-2.79	0.0947	-176:-128
No. Morphemes		all p > .1	
No. Phonemes		all p > .1	
No. PhonSyll	2.9	0.0717	-360:-296
No. PhonSyll	2.99	0.0627	-24:47
No. PhonSyll	3.08	0.0412	136:240
No. PhonSyll	2.58	0.029	256:424
No. PhonSyll	2.7	0.0018	472:1080
No. PhonSyll	2.66	0.0546	1104:1200
No. Syllables		None	
Phon. Neigh. Density	2.59	0.0263	111:248
Phon. Neigh. Density	2.36	0.0594	368:463

Supplementary Table 8. Results of temporal permutation cluster test applied to the sub lexical features relative to word onset.

Feature	$\hat{t}$	p	Window
Adjective	-2.65	0.0214	-160:-16
Coord.Conj	2.49	0.0896	-16:47
Coord.Conj	2.98	0.0381	64:152
Coord.Conj	3.35	0.0075	199:360
Determiner		all p > .1	
Noun	3.32	0.0291	215:327
Noun	3.41	0.007	416:632
Noun	2.59	0.0446	648:759
Pronoun	-3.32	0.0753	656:704
Pronoun	-3.63	0.0008	791:1200
Preposition	-3.43	0.0005	-400:-32
Adverb	2.38	0.088	-352:-288
Adverb	2.5	0.0598	136:215
Adverb	3.03	0.0525	303:376
Adverb	3.25	0.0007	416:767
Adverb	2.49	0.0961	848:904
Adverb	3.07	0.0709	1048:1104
TO		all p > .1	
Verb	2.97	0.0672	64:128
Verb	3.19	0.045	344:424
WH-word		all p > .1	
Ex-There		None	

Supplementary Table 9. Results of temporal permutation cluster test applied to the word class features relative to word onset.

Feature	$\hat{t}$	p	Window
No. Closing Nodes	3.53	0.0144	248:392
No. Closing Nodes	4.09	0.0004	408:696
No. Closing Nodes	3.7	0.0001	712:1176
No. Closing Nodes	-4.43	0.0002	-400:-72
No. Closing Nodes	-3.4	0.026	-55:47
No. Opening Nodes	2.41	0.0787	95:168
No. Opening Nodes	2.77	0.0543	256:335
No. Opening Nodes	3.14	0.0109	376:536
No. Opening Nodes	2.81	0.0876	552:608
Sentence End	4.08	0.0001	248:1128
Sentence End	4.4	0.0579	1144:1200
Sentence End	-2.79	0.0852	-312:-248

Supplementary Table 10. Results of temporal permutation cluster test applied to the syntactic operation features relative to word onset.

Feature	$\hat{t}$	p	Window
Tree Depth	11.17	0.0002	-400:47
Tree Depth	10.06	0.0001	72:1200
Tree Depth -1	10.12	0.0005	-400:-40
Tree Depth -1	9.28	0.045	-24:47
Tree Depth -1	8.6	0.0001	64:1152
Tree Depth -1	8.15	0.0587	1168:1200
Tree Depth +1	10.72	0.0001	-400:47
Tree Depth +1	9.39	0.0001	64:1200
No. Open Nodes	10.65	0.0002	-400:47
No. Open Nodes	9.24	0.0001	64:1200
Linear Order	13.24	0.0001	-400:47
Linear Order	11.29	0.0001	64:1200
Distance from end	11.82	0.0313	-400:-280
Distance from end	10.91	0.0036	-264:0
Distance from end	10.9	0.055	16:47
Distance from end	9.96	0.0174	64:288
Distance from end	9.65	0.0001	303:1200
Linear Order -1	10.69	0.0002	-400:47
Linear Order -1	9.15	0.0001	64:1200
Linear Order +1	11.02	0.0002	-400:47
Linear Order +1	9.92	0.0001	64:1200
Distance from start	12.07	0.0002	-400:47
Distance from start	10.92	0.0001	64:1200

Supplementary Table 11. Results of temporal permutation cluster test applied to the syntactic state features relative to word onset.

Feature	$\hat{t}$	p	Window
GloVe PC1	2.93	0.0063	-400:16
GloVe PC1	4.66	0.0019	64:472
GloVe PC1	4.47	0.0001	488:1200
GloVe PC2	3.2	0.0231	-400:-240
GloVe PC2	3.43	0.0087	-224:47
GloVe PC2	3.13	0.0921	64:111
GloVe PC2	3.65	0.0002	136:968
GloVe PC3	2.62	0.0735	280:352
GloVe PC3	2.73	0.0061	744:1200
GloVe PC4	3.15	0.0354	-400:-272
GloVe PC4	3.39	0.0125	-200:47
GloVe PC4	3.28	0.0005	64:1200
GloVe PC5		all p > .1	
GloVe PC6	2.79	0.0127	-400:-128
GloVe PC6	2.4	0.0779	79:152
GloVe PC6	2.36	0.0421	696:824
GloVe PC6	2.6	0.04	1000:1120
GloVe PC7	2.54	0.0821	791:872
GloVe PC7	2.99	0.0115	888:1200
GloVe PC8	3.6	0.0021	103:376
GloVe PC9		all p > .1	
GloVe PC10		all p > .1	

Supplementary Table 12. Results of temporal permutation cluster test applied to the semantic word embedding features relative to word onset.
